# Supplementary material for: Solution structures of multiple G-quadruplex complexes induced by a platinum(II)-based tripod reveal dynamic binding
Source: Nat Commun. 2018 Aug 29;9:3496. doi: 10.1038/s41467-018-05810-4 (PMC6115404; doi:10.1038/s41467-018-05810-4)
Supplement: Supplementary file 1 — Supplementary Information [file 41467_2018_5810_MOESM1_ESM.pdf]

Supplementary Information

Solution structures of multiple G-quadruplex complexes induced by a platinum(II)-based tripod reveal dynamic binding

Liu *et al.*

Supplementary Figures

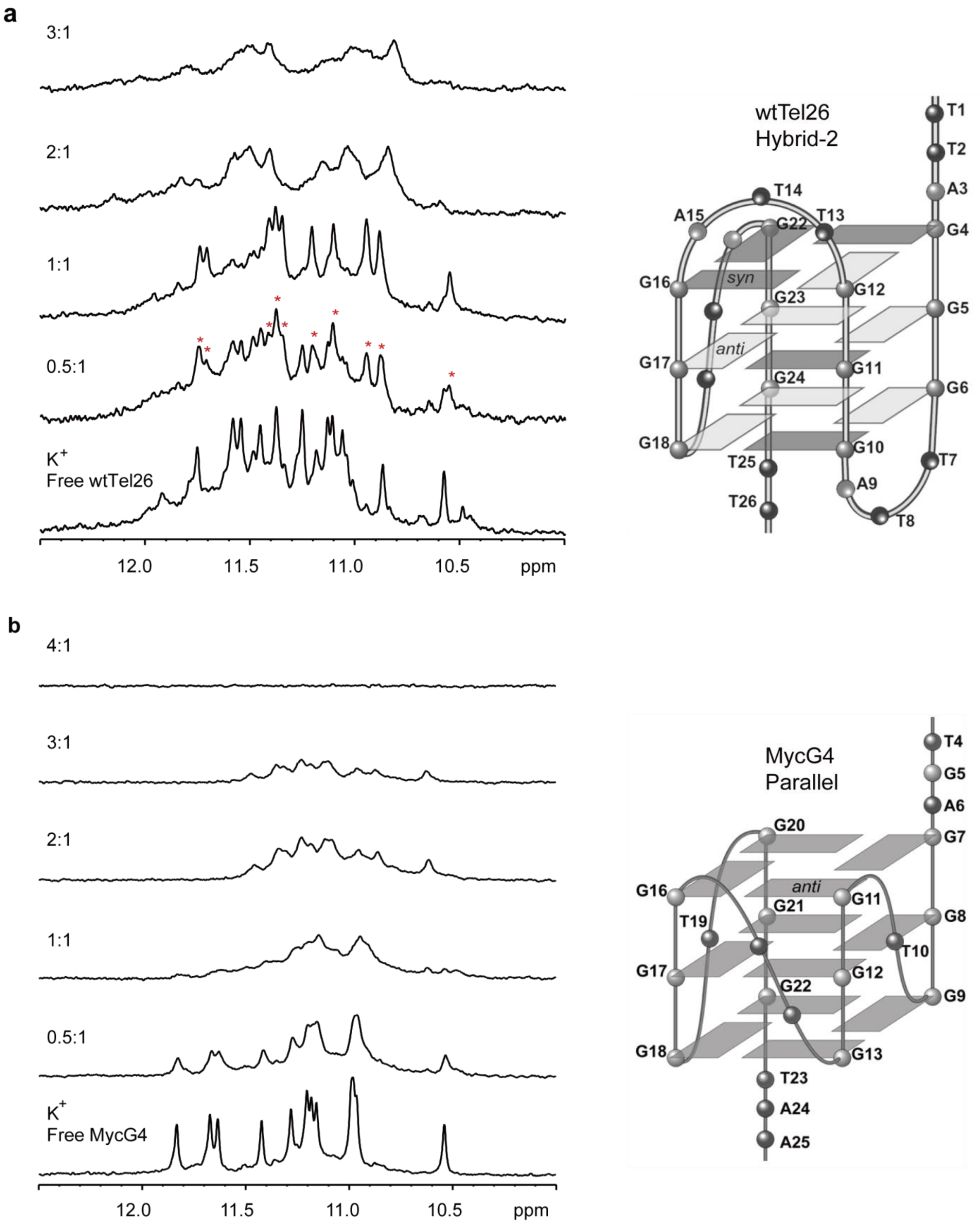

Supplementary Figure 1. The imino proton regions of the 1D ^{1}H NMR titration spectra of Pt-tripod with wtTel26 and MycG4 G-quadruplexes. (a) The hybrid-2 human telomeric G-quadruplex, wtTel26.[3] (b) The major c-Myc G-quadruplex in parallel-stranded.[4] The ratios of Pt-tripod/G-quadruplex are labeled. Condition: pH 7.0, K^+ solution, 25 ℃.

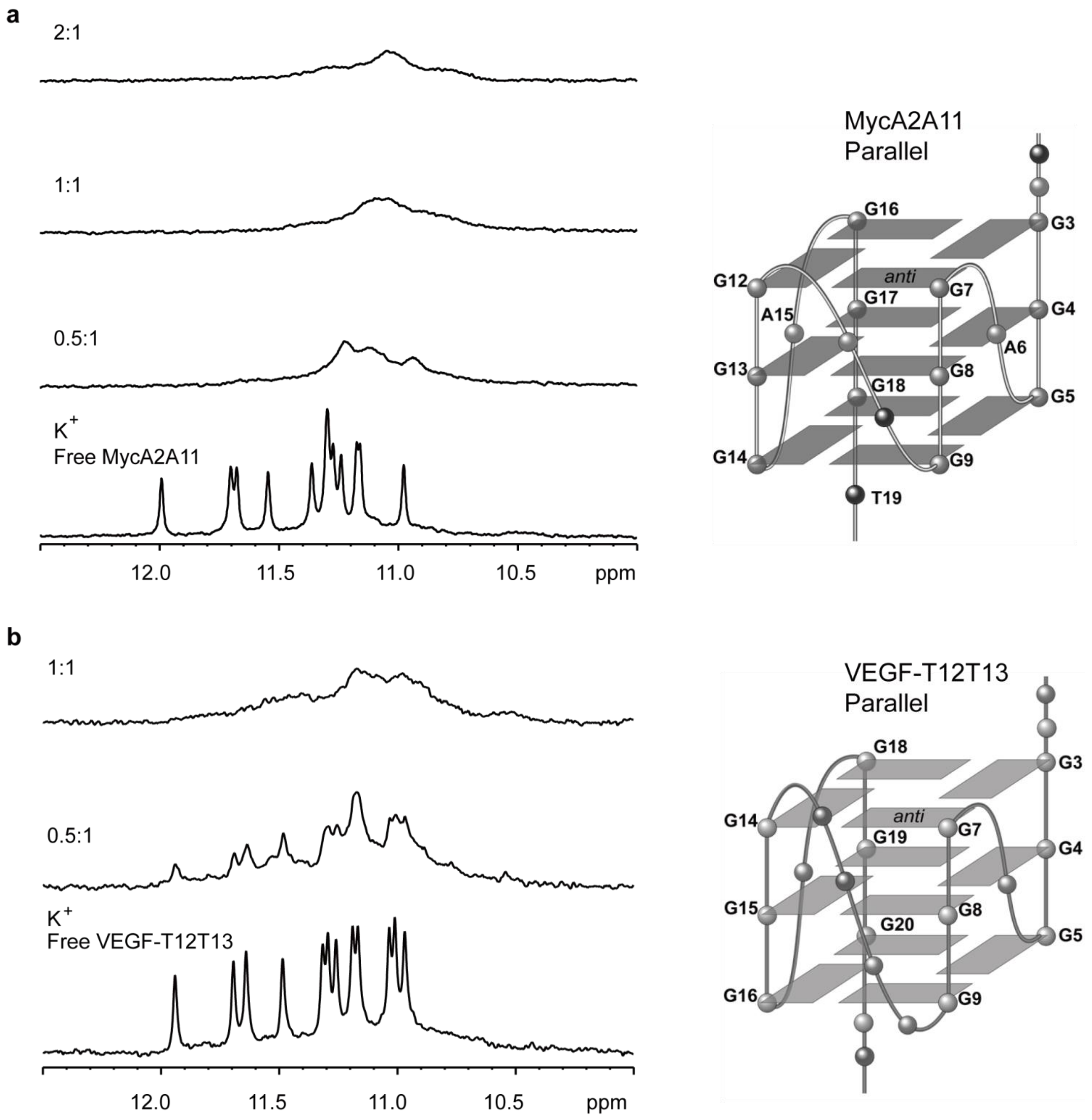

Supplementary Figure 2. The imino proton regions of the 1D ^{1}H NMR titration spectra of Pt-tripod with MycA2A11 and VEGF-T12T13 G-quadruplexes. (a) The parallel-stranded c-Myc G-quadruplexes.[5] (b) The parallel-stranded VEGF G-quadruplex.[6] The ratios of Pt-tripod/G-quadruplex are labeled. Condition: pH 7.0, K^+ solution, 25 ℃.

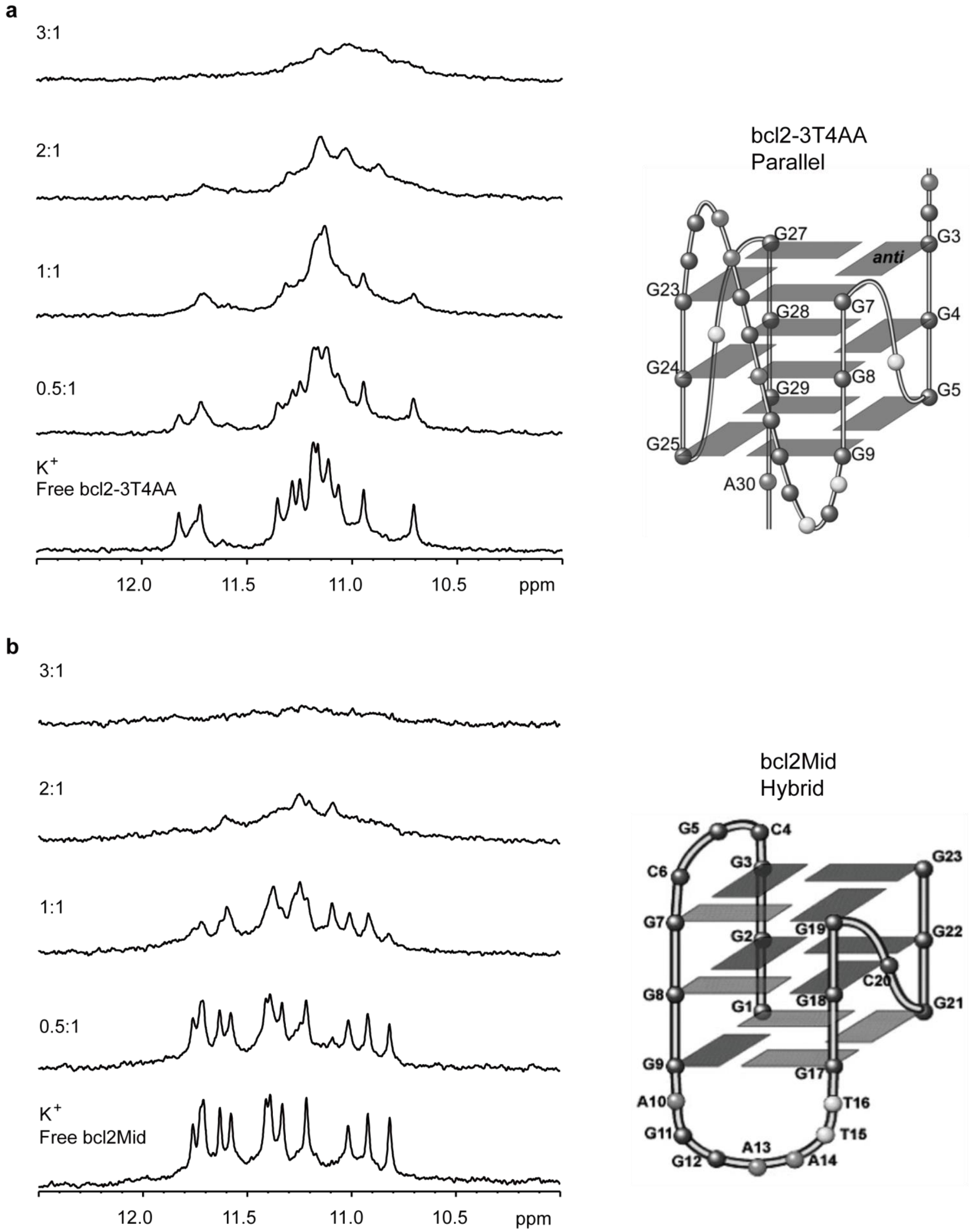

Supplementary Figure 3. The imino proton regions of the 1D ^{1}H NMR titration spectra of Pt-tripod with bcl2-3T4AA and bcl2Mid G-quadruplexes. (a) The parallel-stranded BCL2 G-quadruplex, bcl2-3T4AA.[7] (b) The hybrid BCL2 G-quadruplex, bcl2Mid.[8] The ratios of Pt-tripod/G-quadruplex are labeled. Condition: pH 7.0, K^+ solution, 25 ℃.

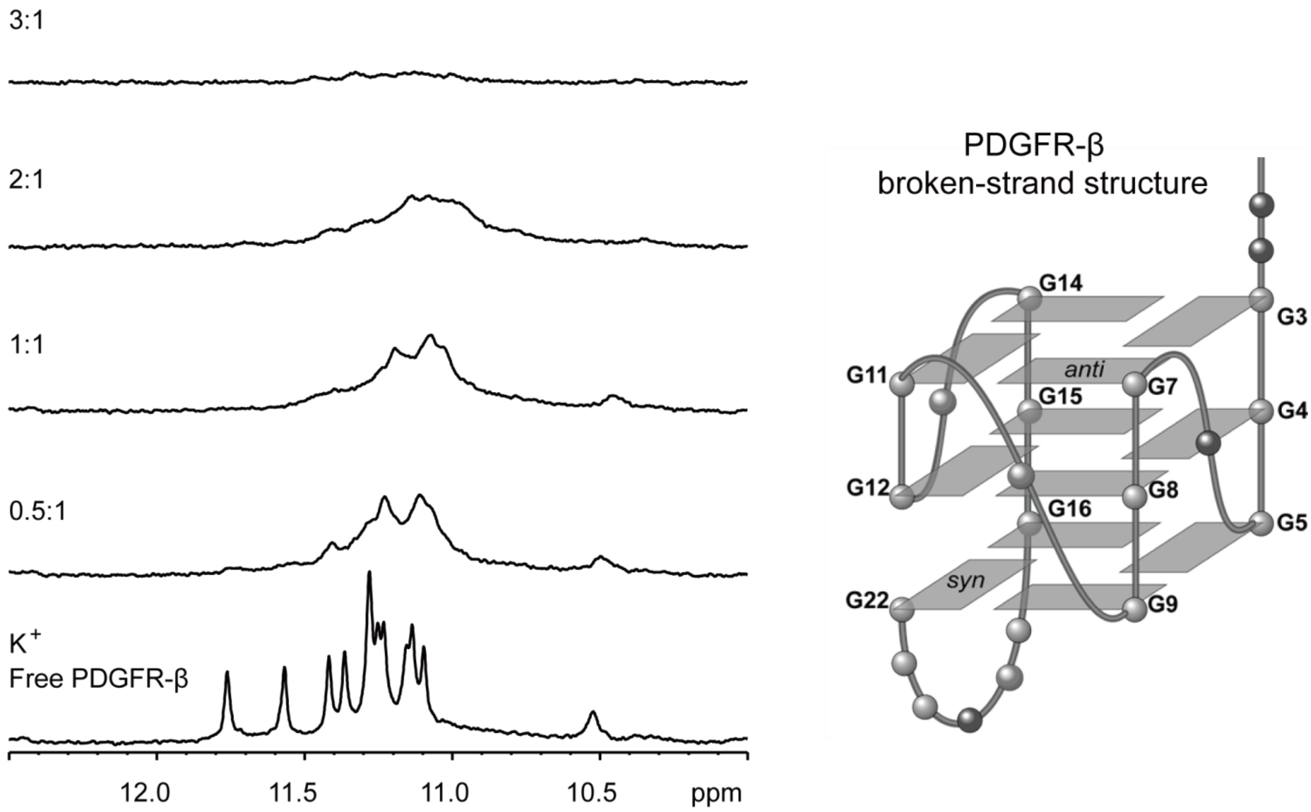

Supplementary Figure 4. The imino proton regions of the 1D ^{1}H NMR titration spectra of Pt-tripod with the broken-stranded PDGFR-β G-quadruplex.[9] The ratios of Pt-tripod/G-quadruplex are labeled. Condition: pH 7.0, K^+ solution, 25 ℃.

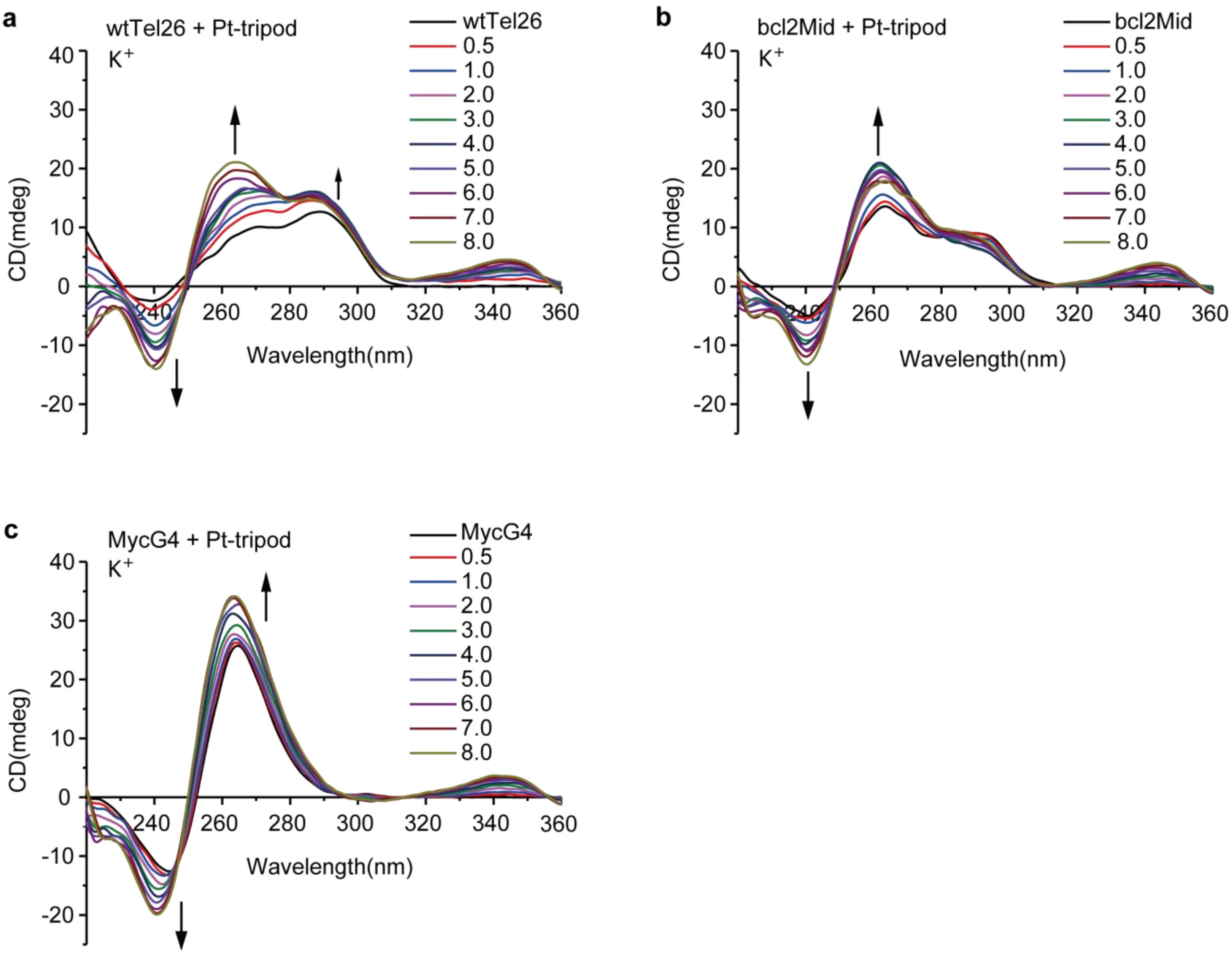

Supplementary Figure 5. CD spectra. The CD titration spectra of Pt-tripod with various G-quadruplexes in 10 mM Tris-HCl, pH 7.4, 100 mM K^+ solution. The ratios of Pt-tripod/G-quadruplex are labeled. The CD spectra illustrate that after interacting with Pt-tripod, the G-quadruplex folding structures remained unchanged.

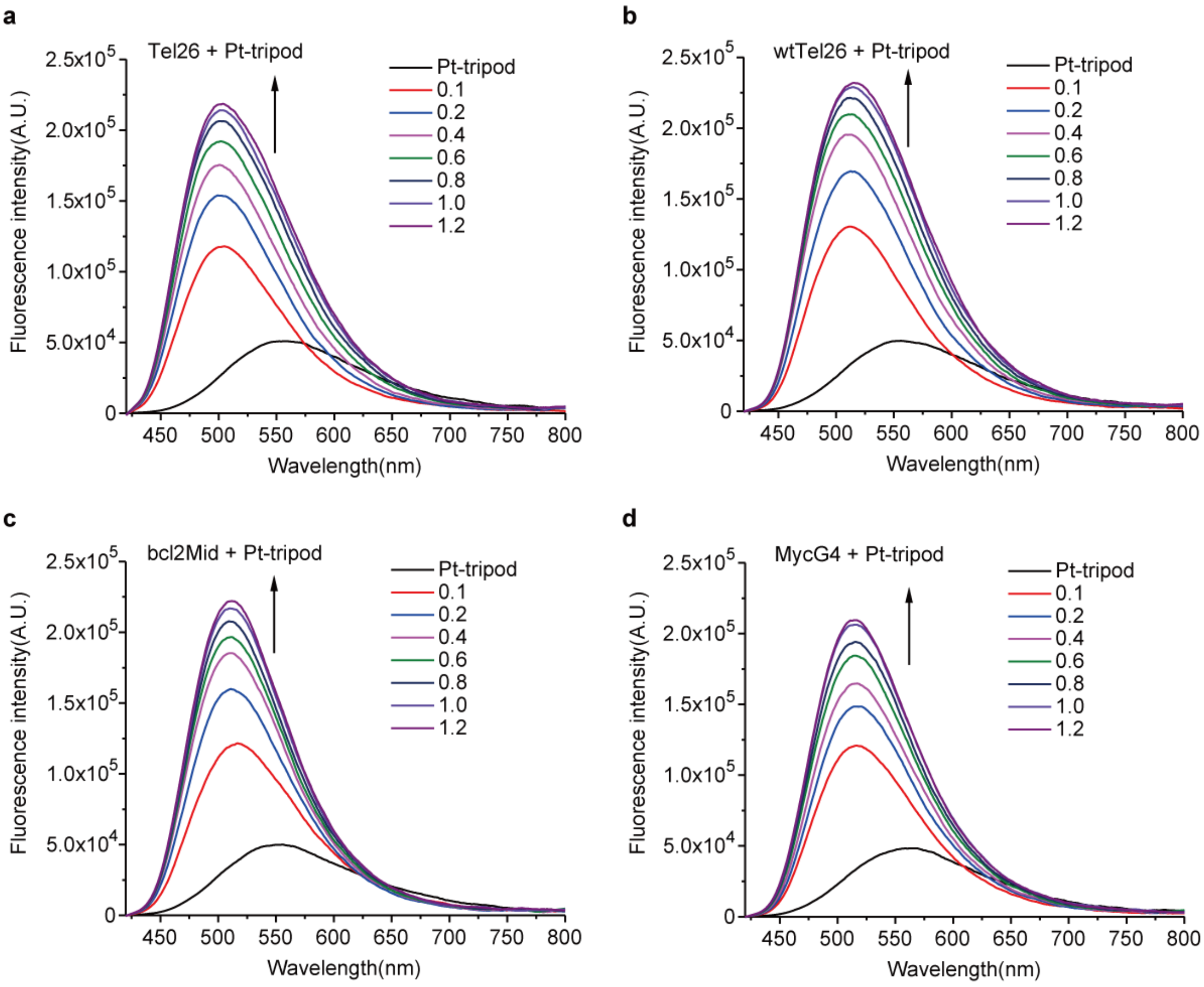

Supplementary Figure 6. Fluorescence emission spectra. The fluorescence emission spectra of the Pt-tripod titrated by different ratios of Tel26 (a), wtTel26 (b), bcl2Mid (c), and MycG4 (d) G-quadruplexes in 10 mM Tris-HCl, 100 mM K^+, pH 7.4. The ratios of G-quadruplex/Pt-tripod are labeled.

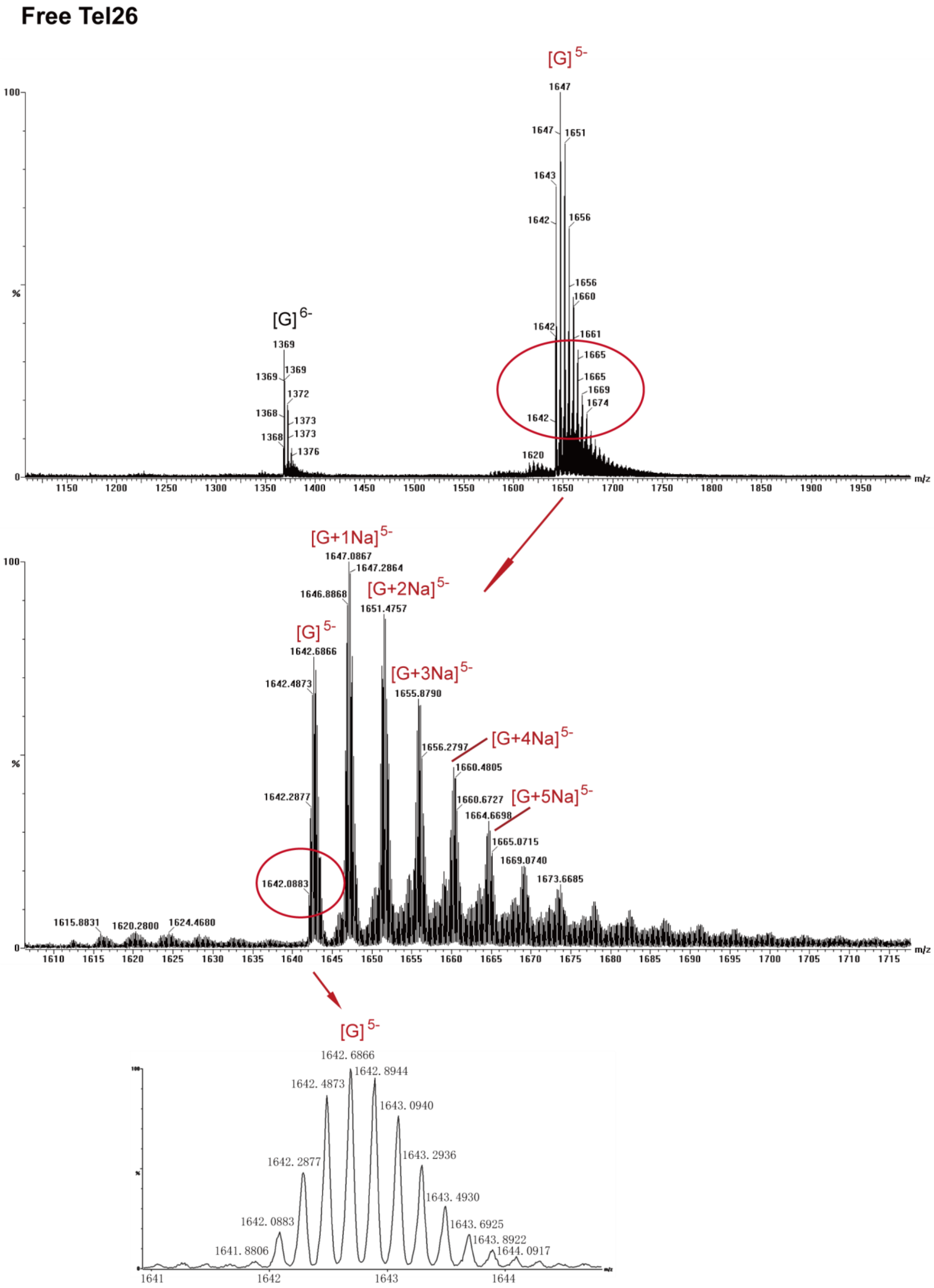

Supplementary Figure 7. The ESI-MS results of the free Tel26 G-quadruplex. Peaks were labeled. ‘G’ means ‘Tel26 Guadruplex’.

1:1

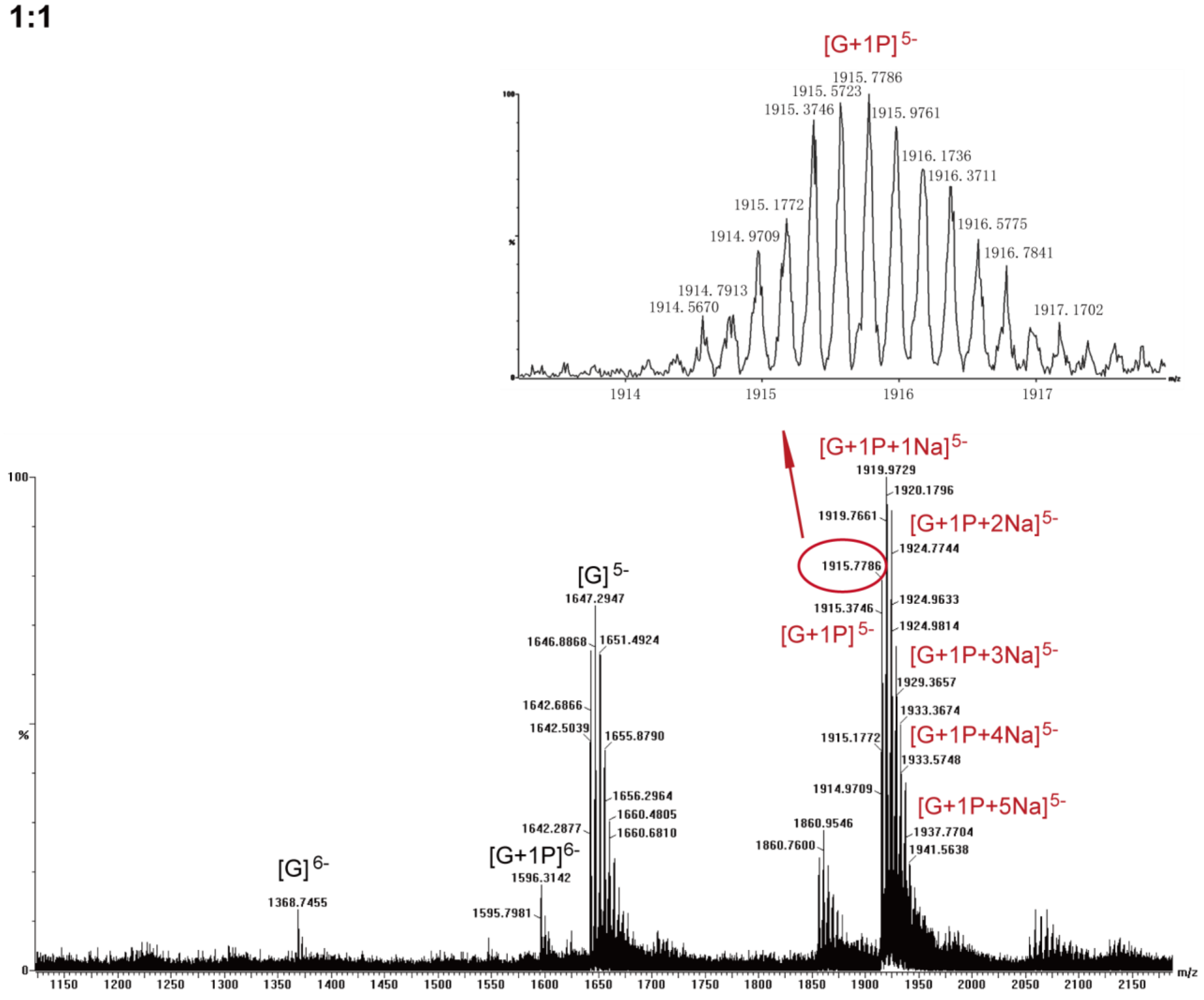

Supplementary Figure 8. The ESI-MS results of the Pt-tripod interacting with the Tel26 G-quadruplex at the Pt-tripod/Tel26 ratio of 1.0. Peaks were labeled. ‘G’ means ‘Tel26 Guadruplex’, and ‘P’ means ‘Pt-tripod’.

2:1

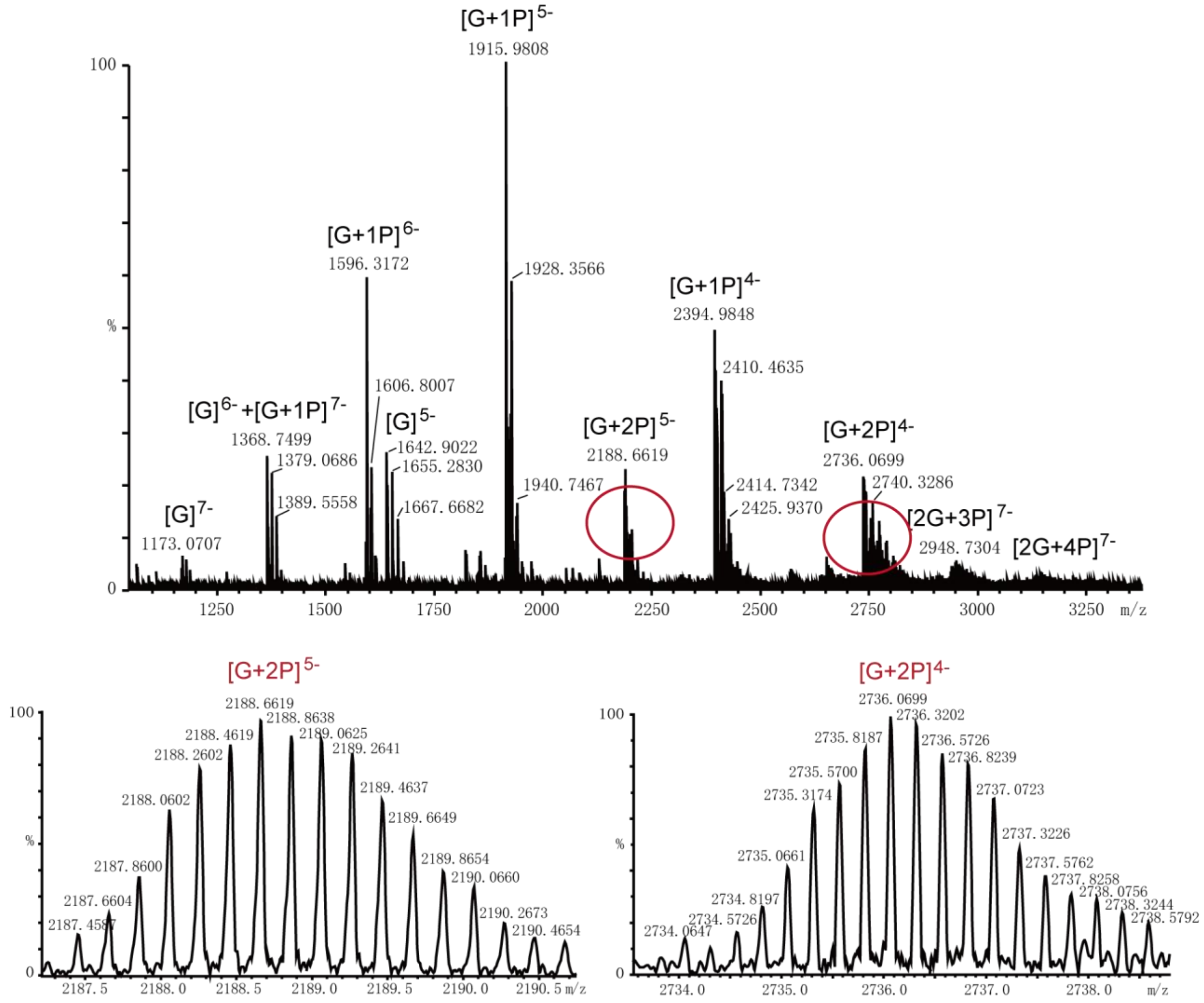

Supplementary Figure 9. The ESI-MS results of the Pt-tripod interacting with the Tel26 G-quadruplex at the Pt-tripod/Tel26 ratio of 2.0. Peaks were labeled. ‘G’ means ‘Tel26 Guadruplex’, and ‘P’ means ‘Pt-tripod’.

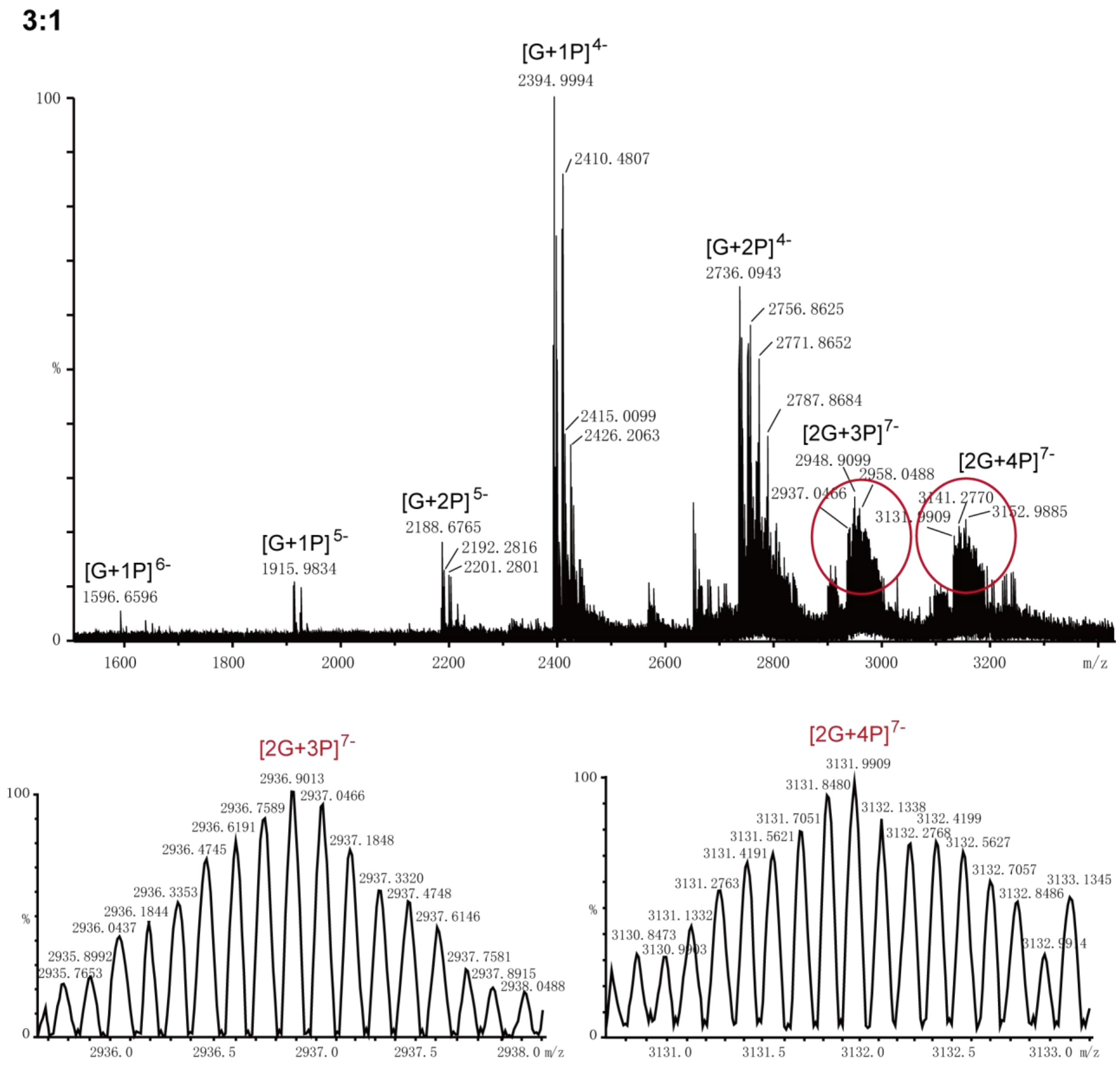

Supplementary Figure 10. The ESI-MS results of the Pt-tripod interacting with the Tel26 G-quadruplex at the Pt-tripod/Tel26 ratio of 3.0. Peaks were labeled. ‘G’ means ‘Tel26 Guadruplex’, and ‘P’ means ‘Pt-tripod’.

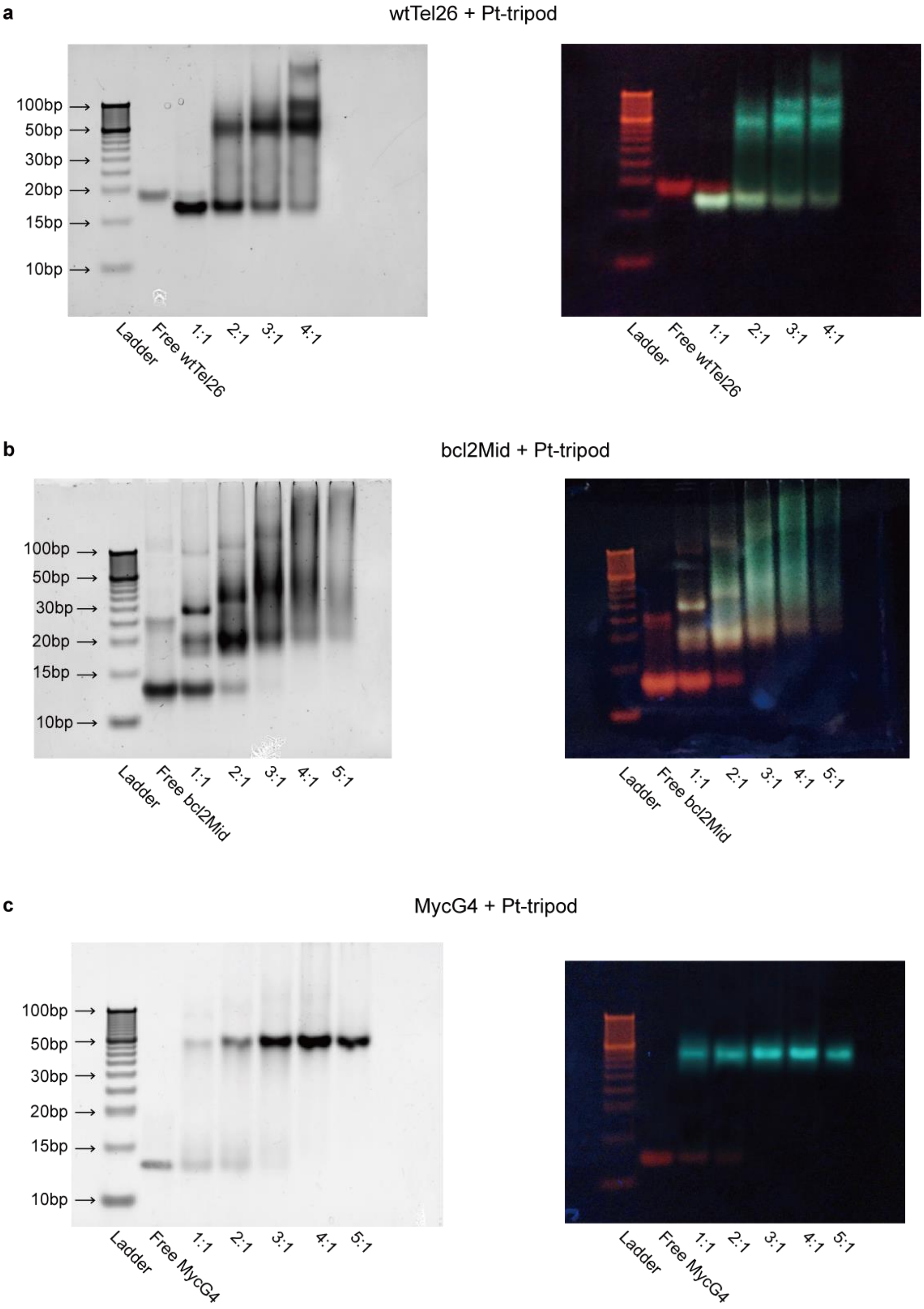

Supplementary Figure 11. Native PAGE experiments show higher-order multimeric structures forming in various G-quadruplexes induced by the Pt-tripod. Native PAGE gel images of wtTel26 (a), bcl2Mid (b) and MycG4 (c) G-quadruplexes interacting with Pt-tripod at different Pt-tripod/DNA ratios, in pH 7.0, K^+ solution. DNA bands were stained by ethidium bromide. The ratios of Pt-tripod/G-quadruplex are labeled. The gel picture was photographed by the FluorChem M imager (ProteinSimple) and visualized by the UV light, respectively.

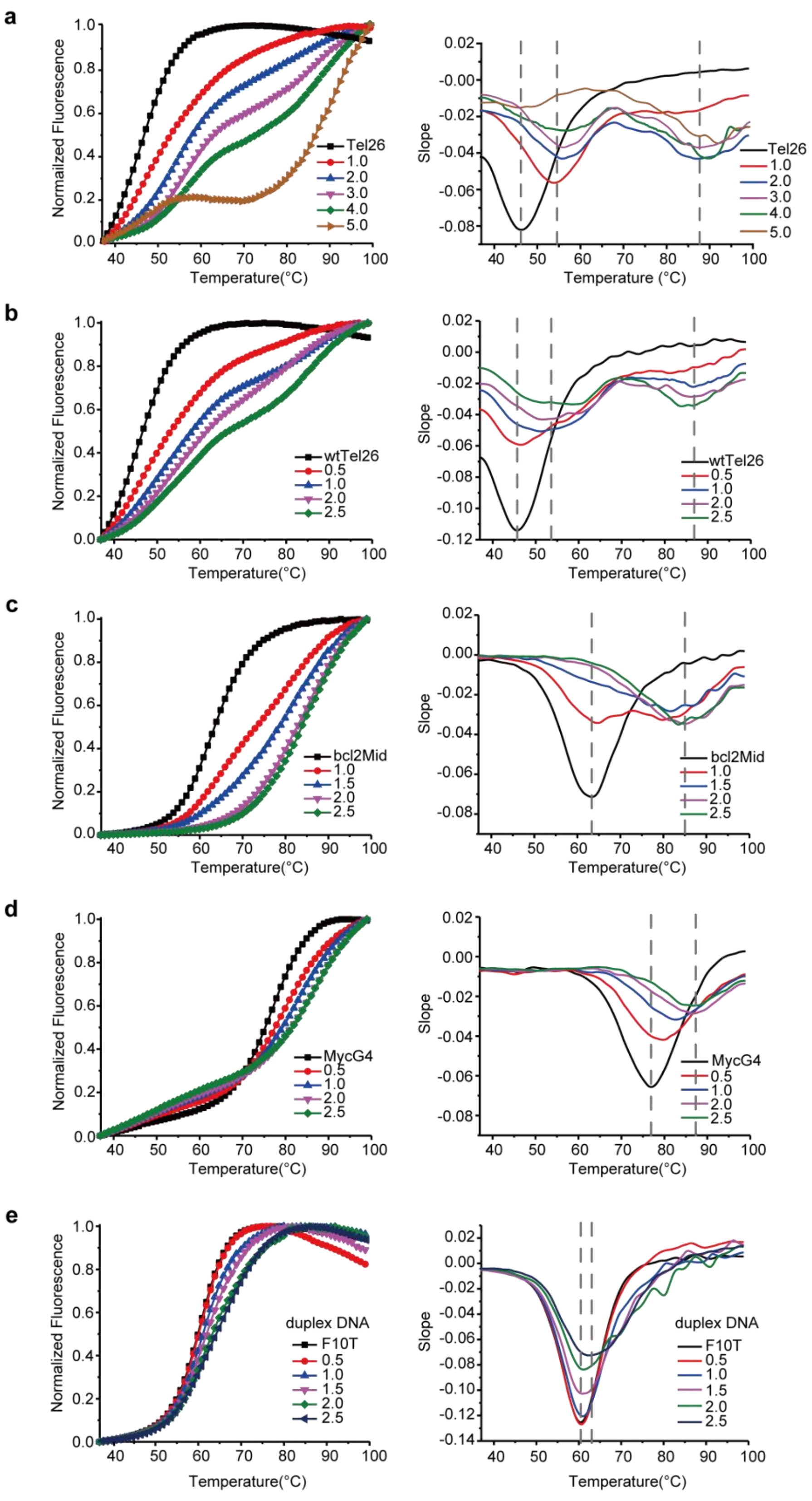

Supplementary Figure 12. FRET melting experiments of the Pt-tripod interacting with various DNA sequences. The FRET melting curves (left) and its slope plots (right) of Tel26 (a), wtTel26 (b), bcl2Mid (c), and MycG4 (d) G-quadruplexes, and duplex DNA F10T (e) interacting with different ratios of Pt-tripod in pH 7.4, 60 mM K^+ solution. The ratios of Pt-tripod/DNA are labeled.

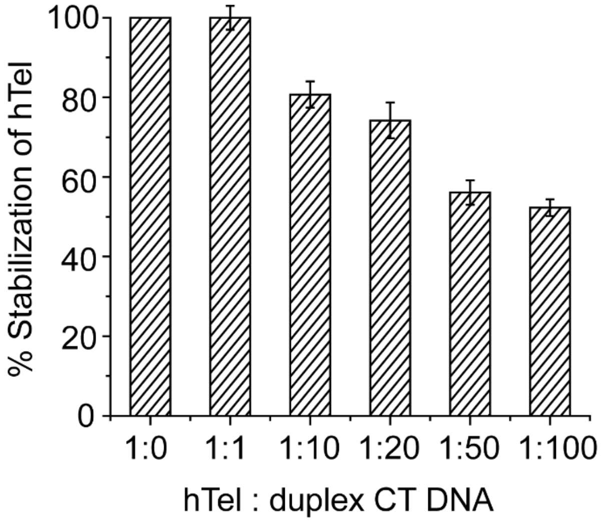

Supplementary Figure 13. FRET competition experiments of the Pt-tripod interacting with human telomeric G-quadruplex and double-stranded DNA. The thermal stabilization ability of Pt-tripod on human telomeric G-quadruplex (hTel) competed with different ratios of double-stranded calf thymus (CT) DNA, using FRET competition assay in pH 7.4, 60 mM K^+ solution. The ratio of Pt-tripod/Tel26 is 1:1. Error bar represents the standard deviation, n = 3.

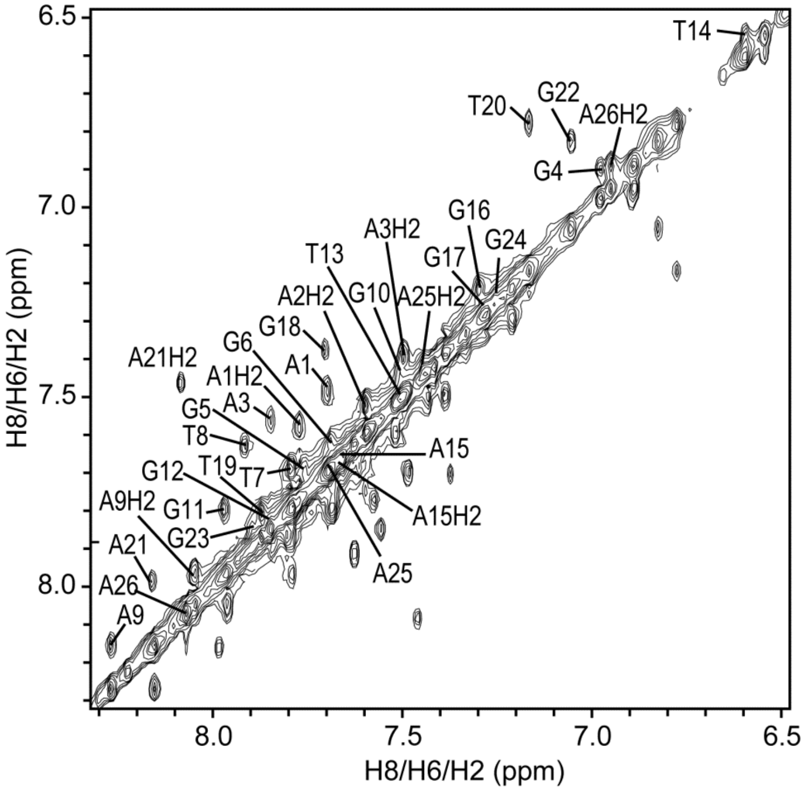

Supplementary Figure 14. The expanded aromatic-aromatic region of the NOESY spectrum at the 0.5:1 Pt-tripod/Tel26 ratio at 25 ℃. Exchange cross-peaks of aromatic protons between free Tel26 and bound Tel26 are labeled with the corresponding residue numbers. The H2 protons are specified.

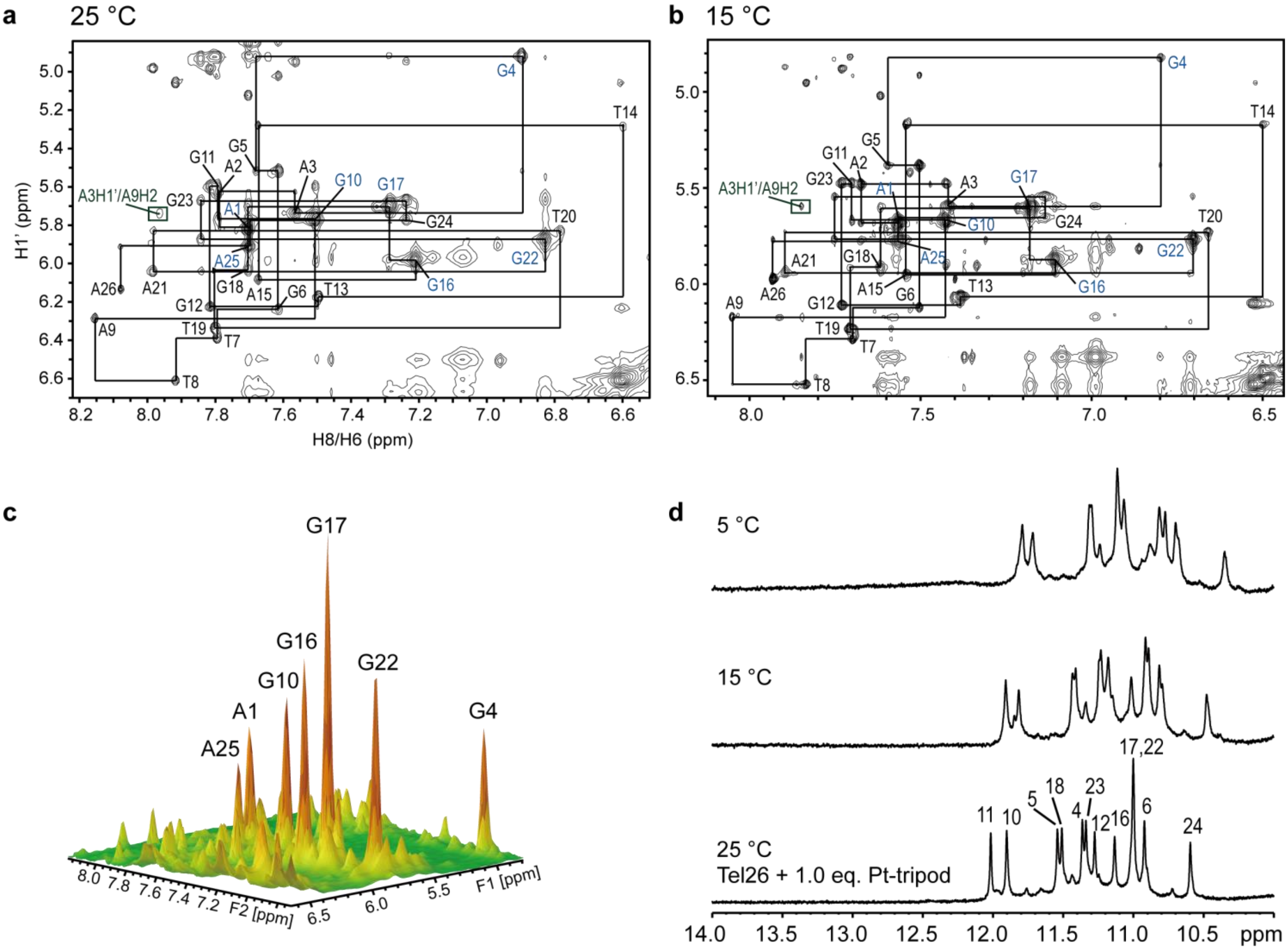

Supplementary Figure 15. NMR studies of the 1:1 Pt-tripod–Tel26 complex in pH 7.0, 100 mM K^+ solution. (a) The expanded H8/H6-H1′ region of the NOESY spectrum of the 1:1 Pt-tripod–Tel26 complex at 25 ℃. (b) The expanded H8/H6-H1′ region of the NOESY spectrum of the 1:1 Pt-tripod–Tel26 complex at 15 ℃. Complete sequential assignment pathway is shown. The H8/H6-H1′ NOEs of the nucleotides in *syn* conformation are labeled in blue, and in *anti* conformation are labeled in black. The inter-residue NOE cross-peak between A3H1′ and A9H2 is framed and labeled in green. (c) Stacked plot of the expanded NOESY spectrum (25 ℃) of the 1:1 Pt-tripod–Tel26 complex. The seven strong intra-residue H8-H1′ NOEs which correspond to the *syn* glycosidic bonds are labeled with residue number. (d) The imino proton regions of the 1D ^{1}H NMR spectrum of the 1:1 Pt-tripod–Tel26 complex at various temperatures of 5, 15, and 25 ℃.

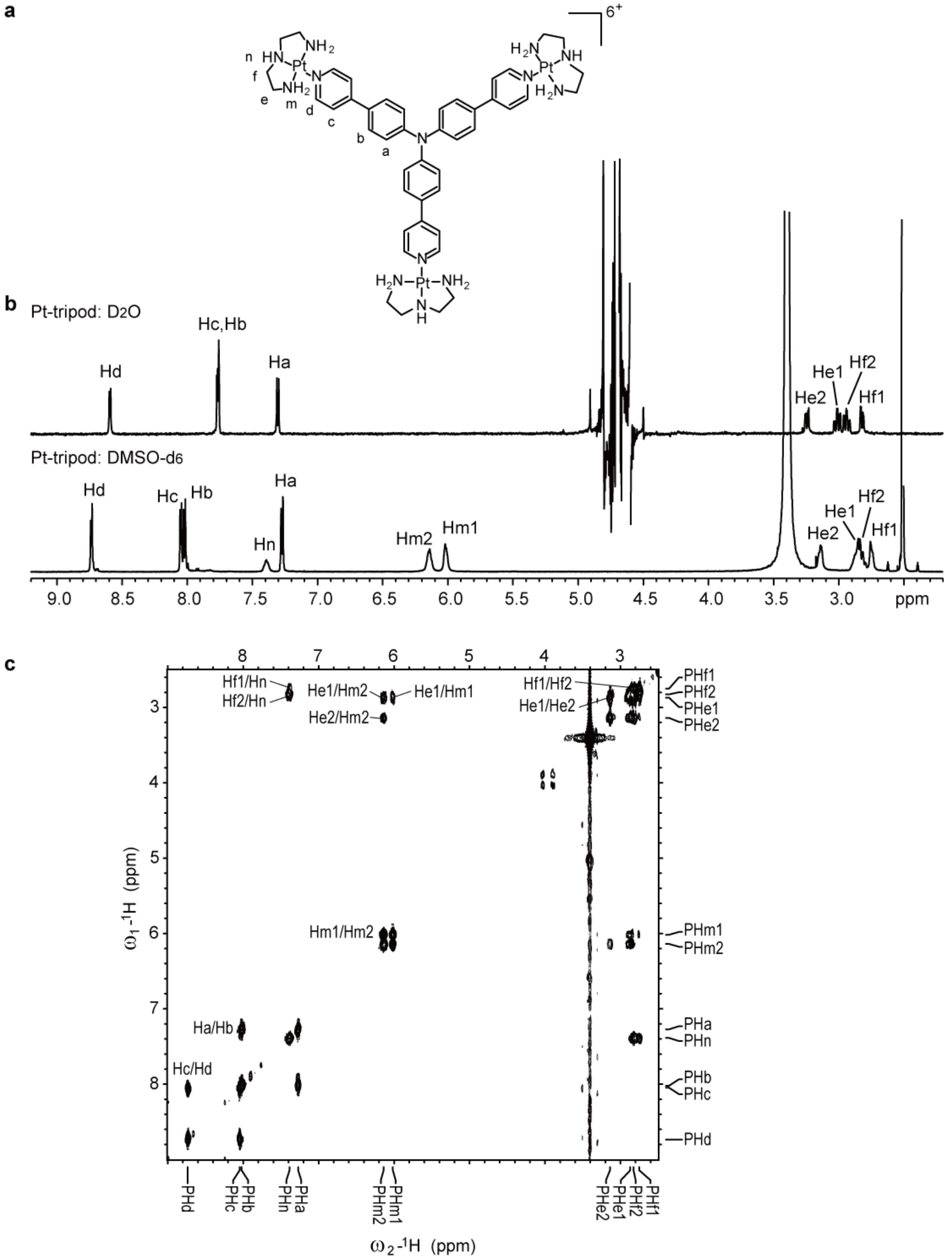

Supplementary Figure 16. NMR assignments of free Pt-tripod. (a) Schematic drawing of Pt-tripod with labeling. (b) The 1D ^{1}H NMR spectrum of the free Pt-tripod in DMSO-d_6 and D_2O at 25 ℃. (c) The expanded COSY spectrum of the free Pt-tripod in DMSO-d_6 at 25 ℃.

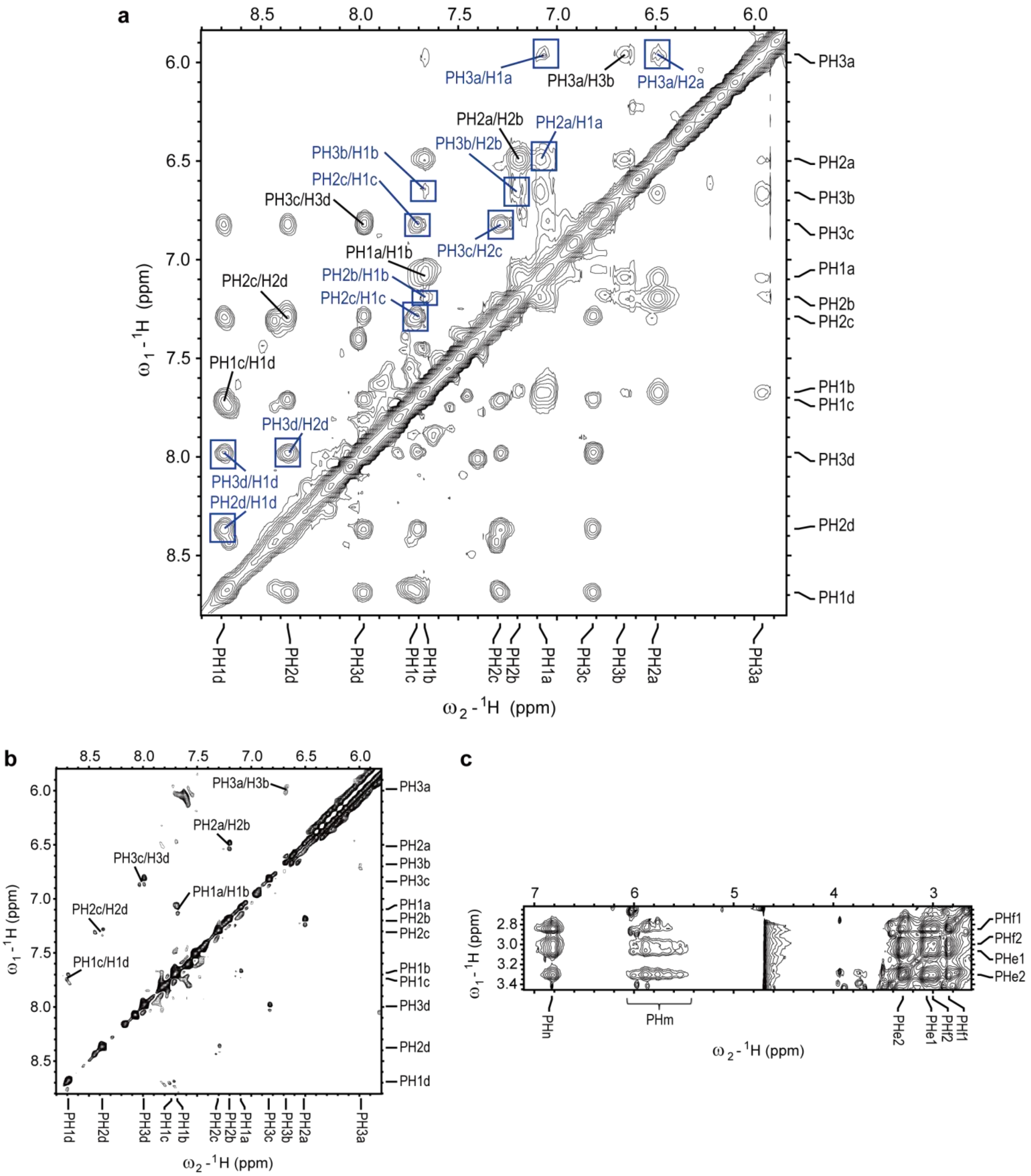

Supplementary Figure 17. 2D NMR assignments of the bound Pt-tripod in the 1:1 Pt-tripod–Tel26 complex. (a) The expanded TOCSY spectrum of the 1:1 Pt-tripod–Tel26 complex showing the assignments of the aromatic regions of bound Pt-tripod. Exchange cross-peaks between the three sets of aromatic NOE peaks of Pt-tripod are labeled in blue. (b) The expanded COSY spectrum of the 1:1 Pt-tripod–Tel26 complex showing the assignments of the aromatic regions of bound Pt-tripod. (c) The expanded NOESY spectrum of the 1:1 Pt-tripod–Tel26 complex showing the assignments of the platinum cationic groups of bound Pt-tripod. Condition: 100 mM K^+, pH 7.0, 25 ℃.

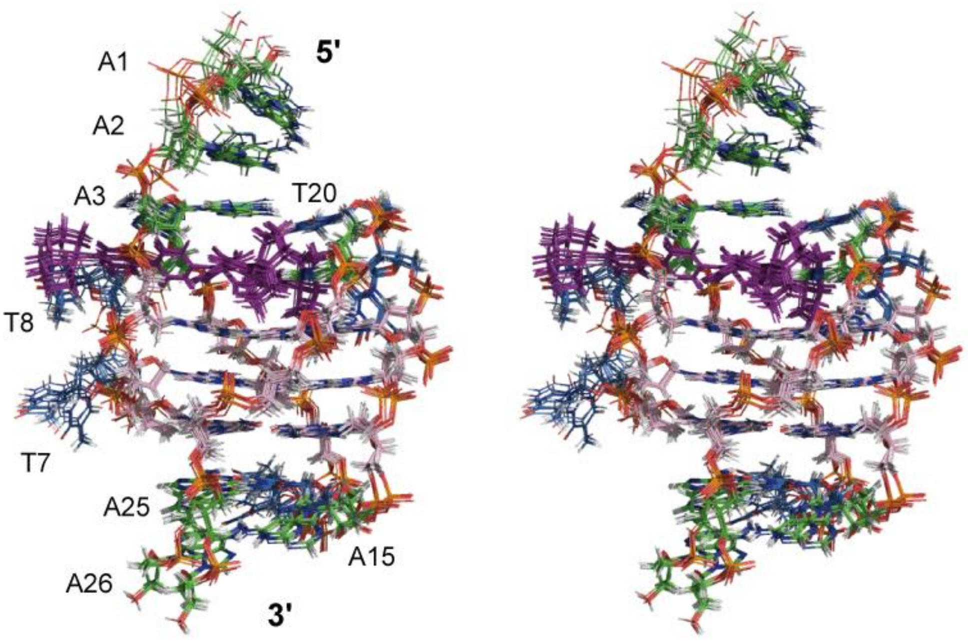

Supplementary Figure 18. The ensemble of the superimposed 15 NMR restraint-refined structures of the 1:1 Pt-tripod–Tel26 complex in stereo view. Pt-tripod molecules are colored in purple. Guanines are colored in pink. Adenines are colored in green. Thymines are colored in blue.

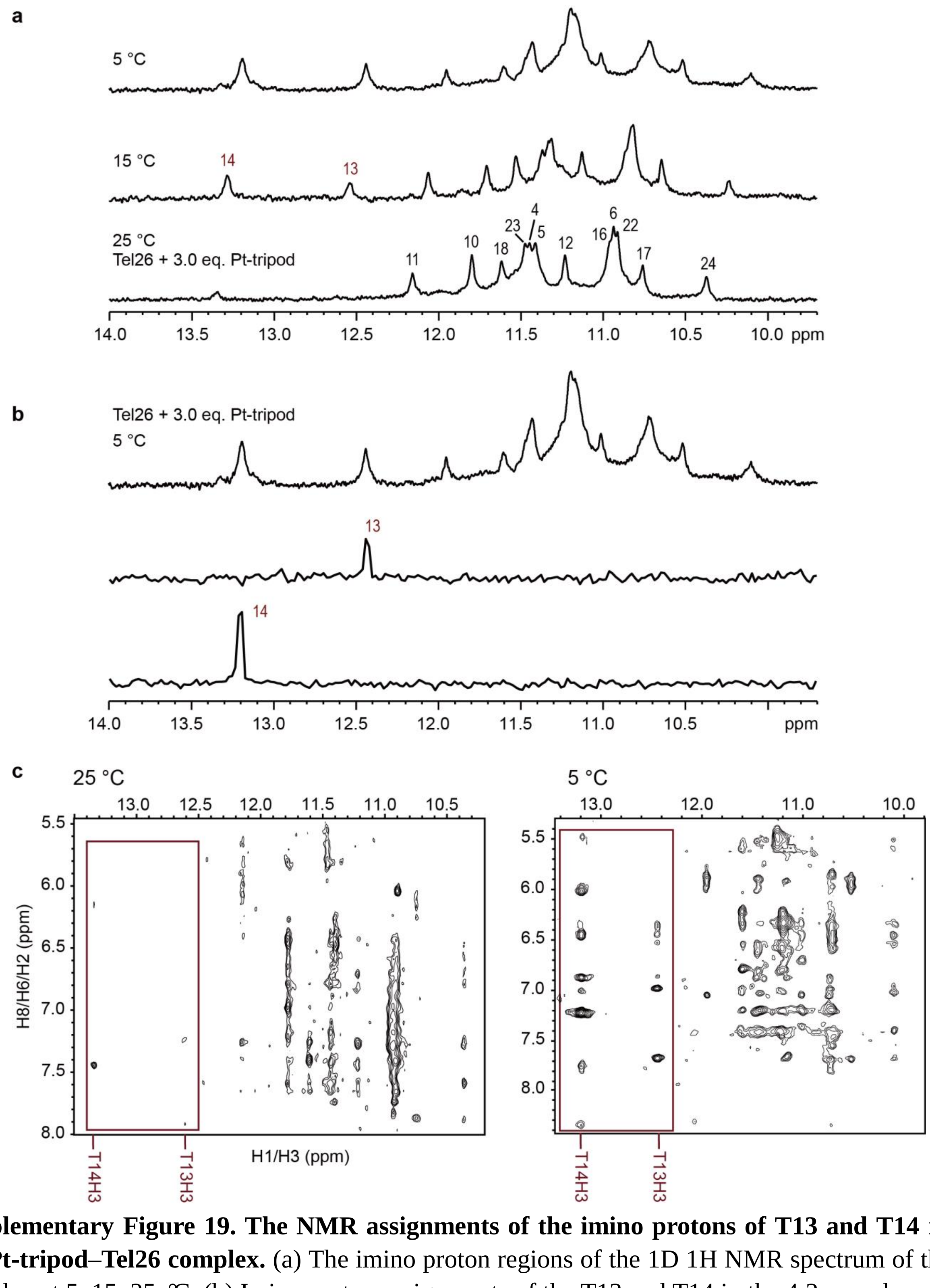

Supplementary Figure 19. The NMR assignments of the imino protons of T13 and T14 in the 4:2 Pt-tripod–Tel26 complex. (a) The imino proton regions of the 1D 1H NMR spectrum of the 4:2 complex at 5, 15, 25 ℃. (b) Imino proton assignments of the T13 and T14 in the 4:2 complex at 5 ℃. The imino proton region of the 1D ^{1}H NMR spectrum of the 4:2 complex with assignments is shown in the top. The imino proton assignments of ^{15}N-filtered experiments are shown below. (c) The expanded imino-imino regions of the NOESY spectra of the 4:2 Pt-tripod–Tel26 complex at 25 ℃ (left) and 5 ℃ (right). Red boxes show the difference between the NOE cross-peaks of T13 and T14 imino protons at different temperatures. Condition: 100 mM K^{+}, pH 7.0.

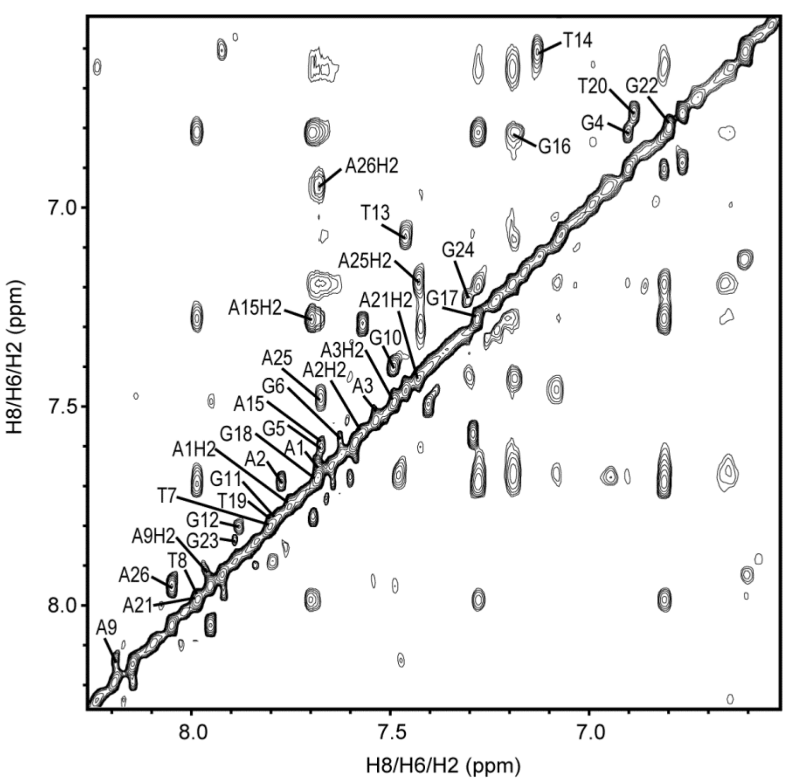

Supplementary Figure 20. The expanded aromatic-aromatic region of the NOESY spectrum at a Pt-tripod/Tel26 ratio of 2.0 at 25 ℃. Exchange cross-peaks of aromatic protons between the 1:1 and 4:2 Pt-tripod–Tel26 complexes are labeled with the corresponding residue numbers. The H2 protons are specified.

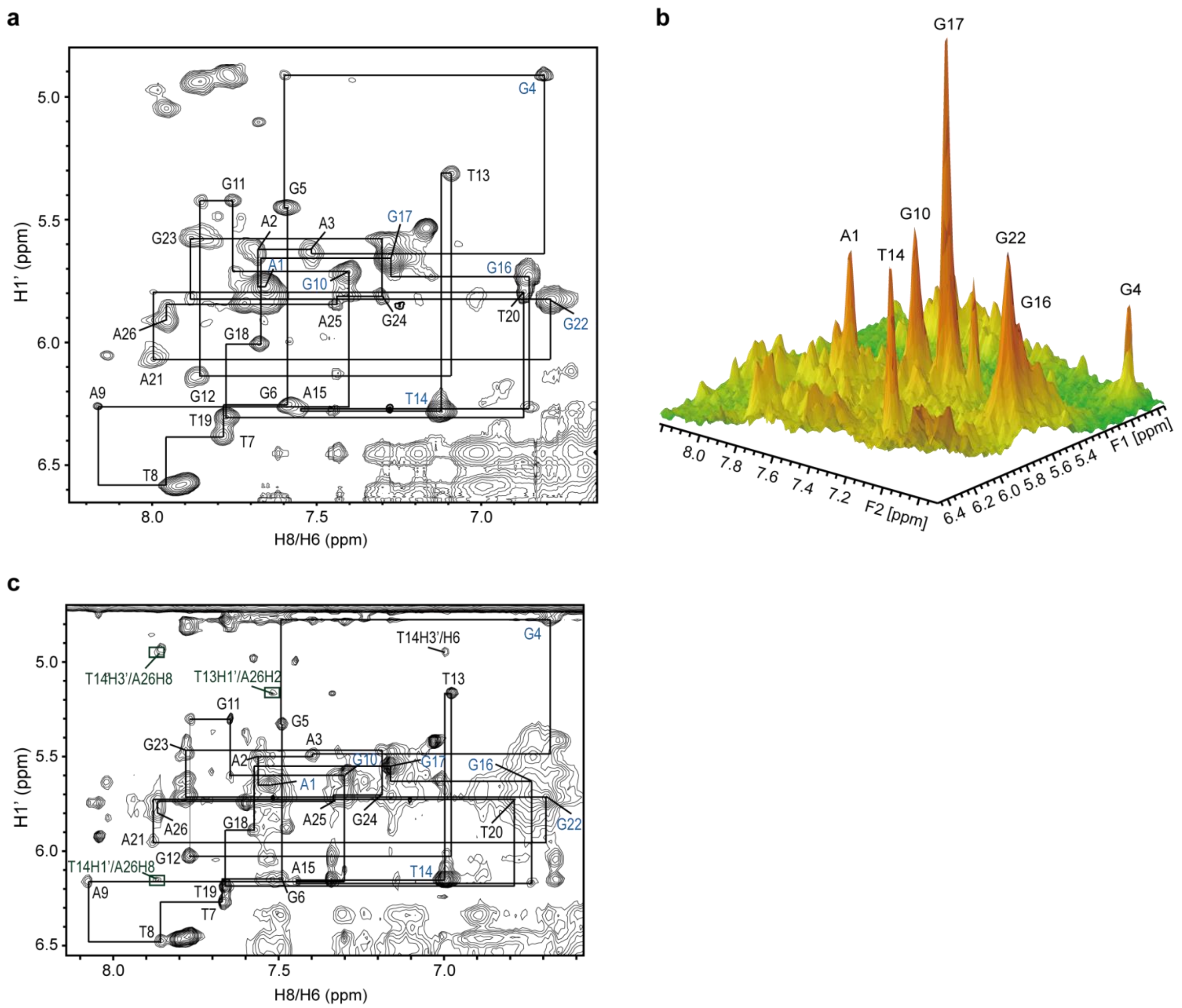

Supplementary Figure 21. NMR studies of the 4:2 Pt-tripod–Tel26 complex in pH 7.0, 100 mM K^+ solution. (a) The expanded H8/H6-H1′ region of the NOESY spectrum of the 4:2 complex in D_2O at 25 ℃. (b) Stacked plot of the expanded NOESY spectrum (25 ℃) of the 4:2 Pt-tripod–Tel26 complex. The seven strong intra-residue H8/H6-H1′ NOEs which correspond to the *syn* glycosidic bonds are labeled with residue number. (c) The expanded H8/H6-H1′ region of the 4:2 complex in H_2O at 15 ℃. Complete sequential assignment pathway is shown. The H8/H6-H1′ NOEs of the nucleotides in *syn* conformation are labeled in blue, and in *anti* conformation are labeled in black. Inter-residue NOEs of T13, T14 and A26 are framed and colored in green.

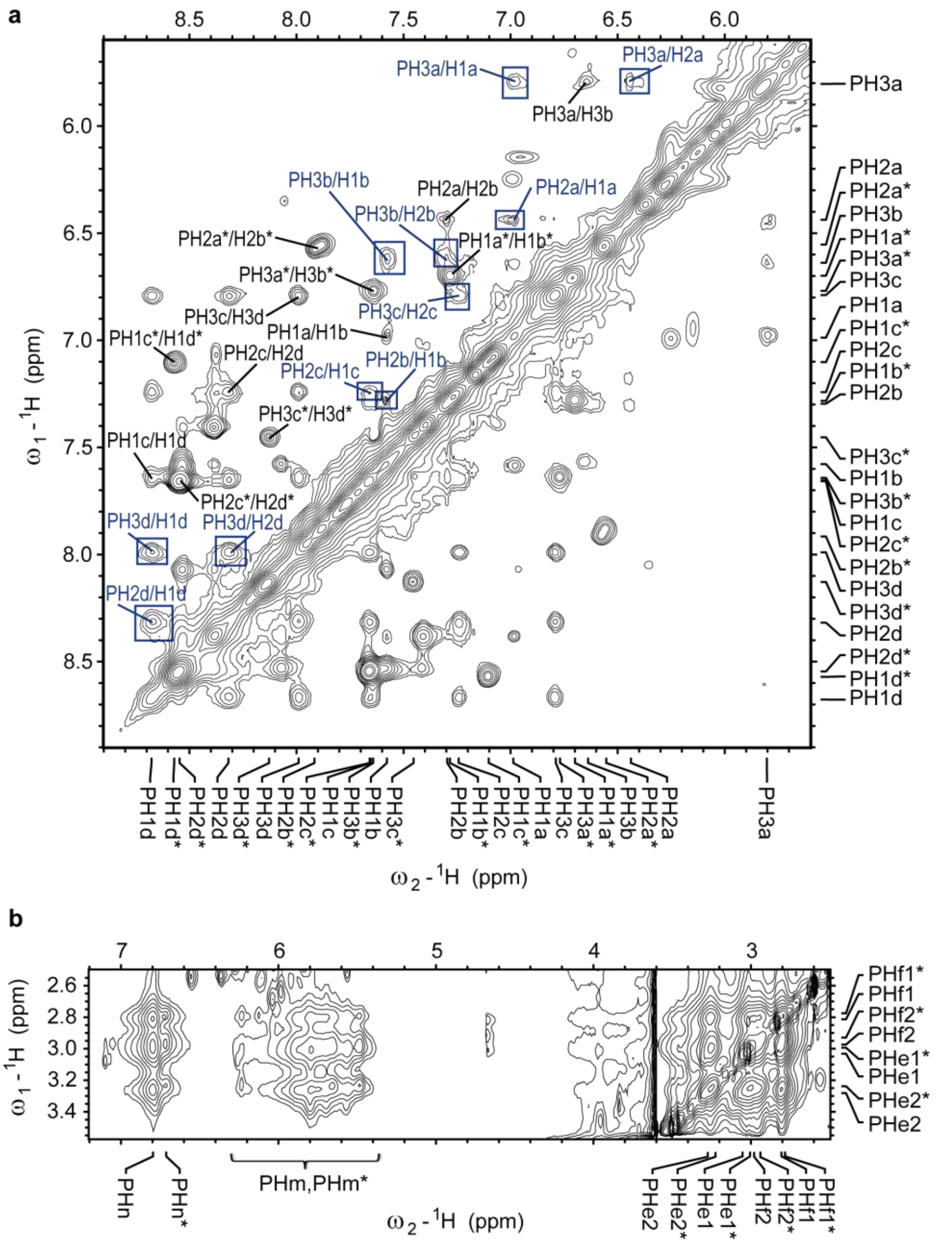

Supplementary Figure 22. 2D NMR assignments of the bound Pt-tripods in the 4:2 Pt-tripod–Tel26 complex. (a) The expanded TOCSY spectrum of the 4:2 complex showing the assignments of the aromatic regions of bound Pt-tripods. Exchange cross-peaks between the three sets of aromatic NOE peaks of the 3'-Pt-tripod are labeled in blue. (b) The expanded NOESY spectrum of the 4:2 complex showing the assignments of the platinum cationic groups of bound Pt-tripod. Assignments belonging to the Pt-tripod bound at the 5′ and 3′ are labeled without or with asterisks. Condition: 100 mM K^+, pH 7.0, 25 ℃.

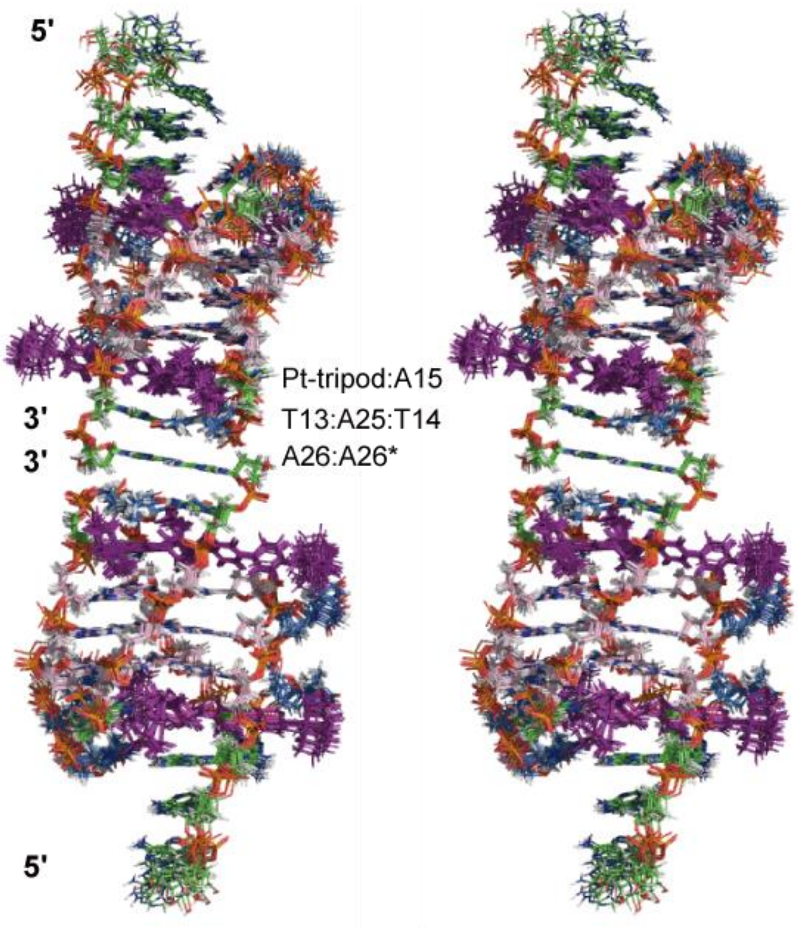

Supplementary Figure 23. The ensemble of the superimposed 15 NMR restraints-refined structures of the dimeric 4:2 Pt-tripod–Tel26 complex in stereo view. The Pt-tripod molecules are colored in purple. Guanines are colored in pink. Adenines are colored in green. Thymines are colored in blue. Residues from the other subunits are labeled with asterisks.

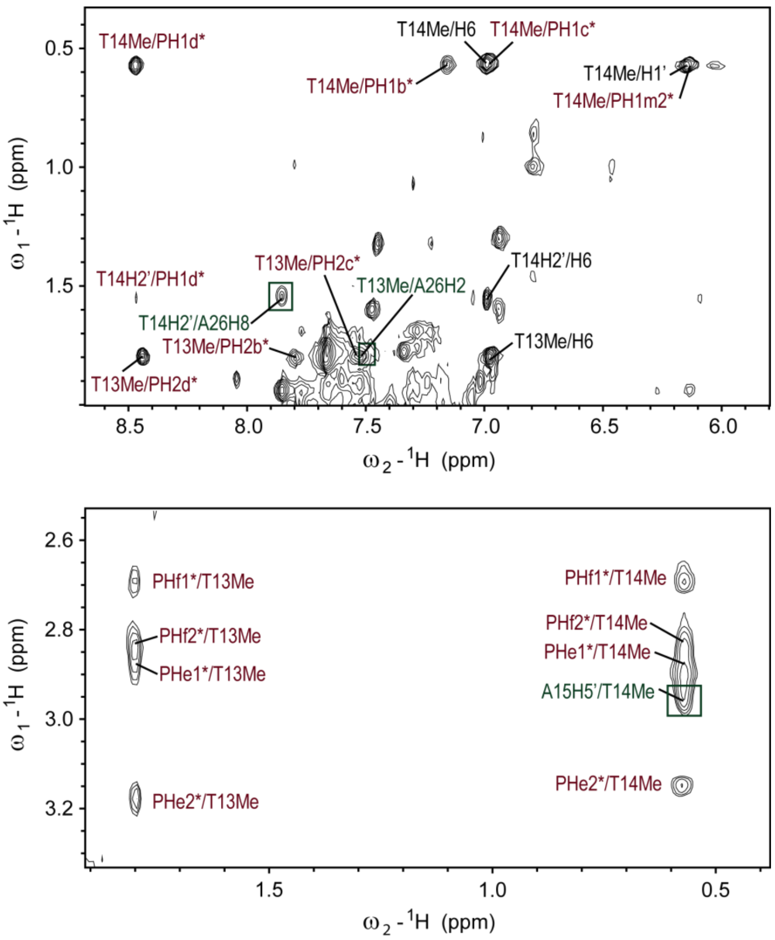

Supplementary Figure 24. The expanded NOESY spectrum of the 4:2 Pt-tripod–Tel26 complex in pH 7.0, 100 mM K^+ solution at 15 ℃. Assignments from the intra-residue NOEs are labeled in black. Assignments from the inter-residue NOEs are framed and labeled in green. Assignments from the intermolecular NOEs between the 3′-Pt-tripod (labeled with asterisks) and 3′-flanking/loop residues are labeled in red.

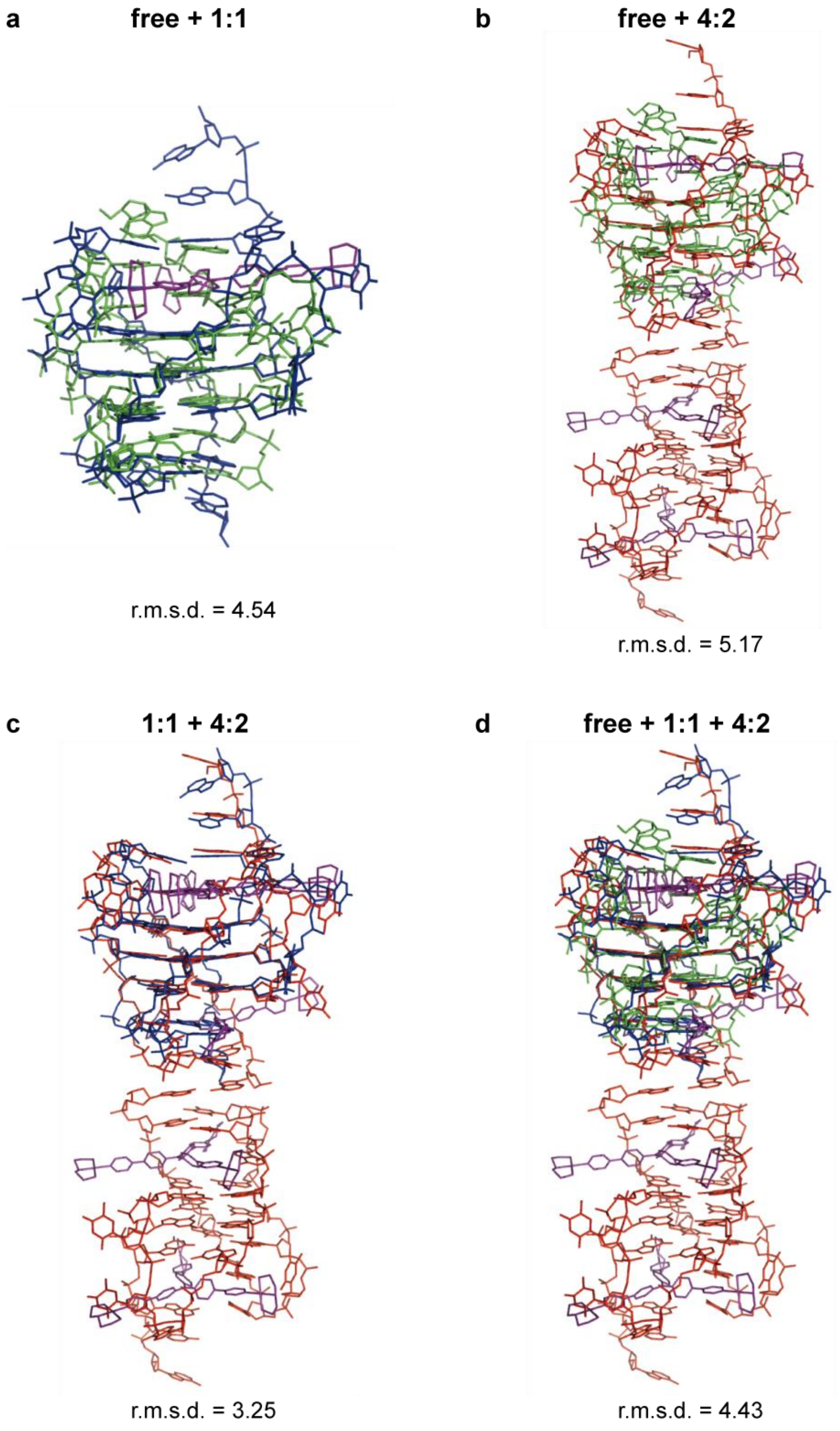

Supplementary Figure 25. Overlapped solution structures of the free Tel26, 1:1 and 4:2 Pt-tripod–Tel26 complexes.

Supplementary Tables

Supplementary Table 1. List of DNA sequences used in this research.[3-10]

Tel26	5′ – AAAGGGTTAGGGTTAGGGTTAGGGAA – 3′
wtTel26	5′ – TTAGGGTTAGGGTTAGGGTTAGGGTT – 3′
MycG4	5′ – TGAGGGTGGGTAGGGTGGGTAA – 3′
MycA2A11	5′ – TAGGGAGGGTAGGGAGGGT – 3′
bcl2Mid	5′ – GGGCGCGGGAGGAATTGGGCGGG – 3′
bcl2-3T4AA	5′ – AGGGGCGGGCGCTTTAGGAAAAGGGCGGGA – 3′
VEGF-T12T13	5′ – CGGGGCGGGCCTTGGGCGGGGT – 3′
PDGFR-β	5′ – AAGGGAGGGCGGCGGGGCAGGG – 3′
TS primer	5′ – AATCCGTCGAGCAGAGTT – 3′
ACX reverse primer	5′ – GCGCGGCTTACCCTTACCCTTACCCTAACC – 3′
TSNT internal control primer	5′ – AATCCGTCGAGCAGAGTTAAAAGGCCGAGAAGCGGAT – 3′
Tel26 (for FRET)	5′ – FAM-AAAGGGTTAGGGTTAGGGTTAGGGAA-TAMRA – 3′
wtTel26 (for FRET)	5′ – FAM-TTAGGGTTAGGGTTAGGGTTAGGGTT-TAMRA – 3′
MycG4 (for FRET)	5′ – FAM-AGGGTTAGGGTTAGGGTTAGGG-TAMRA – 3′
bcl2Mid (for FRET)	5′ – FAM-GGGCGCGGGAGGAATTGGGCGGG-TAMRA – 3′
F10T (for FRET)	5′ – FAM-TATAGCTATA-HEG-TATAGCTATA-TAMRA – 3′

Supplementary Table 2. Melting temperatures (T_m) and stabilization temperatures (ΔT_m) of various DNA with Pt-tripod, obtained from FRET melting experiments.

a. G-quadruplexes

	free DNA	1:1 complex		higher-order complex(es)	
	T_m (℃)	T_m (℃)	ΔT_m (℃)	T_m (℃)	ΔT_m (℃)
Tel26 + Pt-tripod	46.1	54.5	8.4	87.6	41.5
wtTel26 + Pt-tripod	46.1	54.0	7.9	86.8	40.7
Bcl2Mid + Pt-tripod	63.7			85.0	21.3
MycG4 + Pt-tripod	76.7			87.3	10.6

b. Double-stranded DNA

	free DNA	add Pt-tripod	
	T_m (℃)	T_m (℃)	ΔT_m (℃)
F10T + Pt-tripod	60.3	62.8	2.5

Supplementary Table 3. ^{1}H NMR chemical shifts of the 1:1 Pt-tripod–Tel26 complex at 25 ℃.

Tel26 G4	H6/H8	H1/H2 H3/Me	H1'	H2', H2''	H3'	H4'	H5', H5''	NH21, NH22
**A1**	7.70	7.77	5.81	2.03, 2.24	4.60	4.00	3.91, 3.51	
**A2**	7.79	7.60	5.63	2.24	4.75	4.17	3.84, 4.12	
**A3**	7.56	7.50	5.73	2.29, 2.14	4.94	4.29	4.12	
**G4**	6.89	11.36	4.91	3.15, 2.54	4.61	3.97	3.68, 3.44	7.19, –
**G5**	7.68	11.54	5.51	2.44, 2.52	4.83	4.08	3.50, 3.97	6.57, 9.75
**G6**	7.61	10.92	6.23	2.40, 2.47	5.02	4.07	4.01, 4.02	7.51, –
**T7**	7.80	1.93	6.38	2.25, 2.55	4.76	4.46	4.23, 4.07	
**T8**	7.91	2.07	6.60	2.56	5.05	4.61	4.23, 4.07	
**A9**	8.15	7.96	6.28	2.17, 2.24	4.80	4.48	4.12	
**G10**	7.51	11.90	5.76	2.77, 3.29	4.76	4.29	3.96, 4.19	6.55, –
**G11**	7.79	12.01	5.59	2.43, 2.54	4.92	4.30	4.19	5.98, 9.95
**G12**	7.81	11.27	6.22	2.58, 2.52	4.98	4.47	4.15, 4.20	7.85, –
**T13**	7.50	1.79	6.17	2.30, 2.41	4.65	4.15	4.01, 4.09	
**T14**	6.60	1.24	5.27	1.26, 2.06	4.42	3.90	3.51, 3.73	
**A15**	7.67	7.67	6.08	2.16, 2.45	4.59	3.94	2.76, 3.27	
**G16**	7.21	11.13	5.97	3.41, 2.89	4.76	4.38	4.20, 4.26	7.16, –
**G17**	7.29	11.00	5.70	2.36	4.91	4.19	4.10, 4.14	6.46, 8.64
**G18**	7.70	11.51	6.02	2.65	5.12	4.40	4.09, 4.13	
**T19**	7.80	1.91	6.33	2.36, 2.49	4.91	4.39	4.18, 4.27	
**T20**	6.78	1.01	5.82	0.74, 1.57	4.57	4.08	3.88	
**A21**	7.98	7.46	6.04	2.67, 2.87	4.97	4.34	3.74, 3.98	
**G22**	6.82	11.00	5.86	2.80, 3.42	4.92	4.58	4.09, 4.16	7.06, –
**G23**	7.84	11.34	5.66	2.50, 2.57	4.94	4.33	4.10, 4.15	5.82, 9.45
**G24**	7.23	10.59	5.76	1.97, 2.35	4.81	4.27	4.13	7.09, –
**A25**	7.70	7.45	5.90	2.42	4.84	4.18	3.99, 4.02	
**A26**	8.08	6.96	6.13	2.56, 2.37	4.55	4.09	4.00	

<table>
<tr><td rowspan="6">Pt-tripod</td><th>H1a</th><th>H1b</th><th>H1c</th><th>H1d</th><th>H1m1</th><th>H1m2</th><th>He1</th><th>He2</th><th>Hf1</th><th>Hf2</th><th>Hn</th></tr>
<tr><td>7.07</td><td>7.67</td><td>7.70</td><td>8.69</td><td>5.88</td><td>6.00</td><td rowspan="5">3.06</td><td rowspan="5">3.31</td><td rowspan="5">2.84</td><td rowspan="5">2.99</td><td rowspan="5">6.81</td></tr>
<tr><th>H2a</th><th>H2b</th><th>H2c</th><th>H2d</th><th>H2m1</th><th>H2m2</th></tr>
<tr><td>6.49</td><td>7.20</td><td>7.28</td><td>8.37</td><td>5.65</td><td>5.76</td></tr>
<tr><th>H3a</th><th>H3b</th><th>H3c</th><th>H3d</th><th>H3m1</th><th>H3m2</th></tr>
<tr><td>5.96</td><td>6.66</td><td>6.83</td><td>7.99</td><td>5.48</td><td>5.53</td></tr>
</table>

Supplementary Table 4. ^{1}H NMR chemical shifts of the 1:1 Pt-tripod–Tel26 complex at 15 ℃.

Tel26 G4	H6/H8	H1/H2 H3/Me	H1'	H2', H2''	H3'	H4'	H5', H5''	NH21, NH22
**A1**	7.56	7.64	5.68	1.91, 2.14	4.48	3.90	3.81, 3.39	
**A2**	7.67	7.47	5.48	2.14	4.64	4.06	3.74, 4.03	
**A3**	7.42	7.35	5.59	2.16, 2.02	4.85	4.19	4.04	
**G4**	6.80	11.24	4.80	3.05, 2.43	4.48	3.78	3.60, 3.35	6.48, 8.82
**G5**	7.60	11.43	5.38	2.34, 2.40	4.73	3.95	3.37, 3.85	6.46, 9.67
**G6**	7.50	10.81	6.12	2.28, 2.36	4.91	3.95	3.89	7.48, –
**T7**	7.70	1.82	6.28	2.12, 2.44	4.65	4.36	4.12, 3.95	
**T8**	7.83	1.97	6.52	2.46	4.95	4.52	4.14, 3.95	
**A9**	8.05	7.85	6.17	2.11	4.69	4.38	4.02, 4.00	
**G10**	7.43	11.81	5.67	2.67, 3.19	4.65	4.18	3.82, 4.08	6.55, –
**G11**	7.70	11.90	5.48	2.33, 2.42	4.82	4.18	4.09	5.87, 9.92
**G12**	7.73	11.17	6.11	2.48, 2.41	4.88	4.37	4.11, 4.08	7.56, 8.83
**T13**	7.38	1.68	6.07	2.19, 2.30	4.55	4.04	3.91, 3.99	
**T14**	6.50	1.12	5.17	1.15, 1.96	4.31	3.80	3.41, 3.62	
**A15**	7.54	7.54	5.95	2.05, 2.35	4.47	3.81	2.66, 3.15	
**G16**	7.11	11.01	5.87	3.31, 2.79	4.66	4.27	4.09, 4.17	7.07, –
**G17**	7.18	10.89	5.60	2.25	4.81	4.09	3.95, 4.02	5.96, 9.43
**G18**	7.62	11.41	5.91	2.57, 2.54	5.02	4.30	4.02, 4.09	
**T19**	7.71	1.80	6.23	2.26, 2.38	4.82	4.28	4.08, 4.15	
**T20**	6.66	0.88	5.73	0.65, 1.50	4.48	3.97	3.75, 3.79	
**A21**	7.90	7.37	5.94	2.77, 2.57	4.87	4.24	3.63, 3.87	
**G22**	6.70	10.91	5.77	3.33, 2.70	4.82	4.48	3.98, 4.04	6.60, 8.64
**G23**	7.75	11.23	5.55	2.40, 2.45	4.84	4.22	3.99, 4.04	5.65, 9.41
**G24**	7.14	10.47	5.65	1.88, 2.24	4.72	4.17	4.00, 4.02	5.72, –
**A25**	7.56	7.31	5.77	2.24, 2.30	4.74	4.08	3.87, 3.91	
**A26**	7.93	6.86	5.97	2.43, 2.23	4.42	3.97	3.89, 3.91	

							He1	He2	Hf1	Hf2	Hn
**Pt-tripod**	**H1a**	**H1b**	**H1c**	**H1d**	**H1m1**	**H1m2**	2.93	3.19	2.72	2.88	6.73
	6.99	7.58	7.61	8.58	5.80	5.93					
	**H2a**	**H2b**	**H2c**	**H2d**	**H2m1**	**H2m2**					
	6.38	7.09	7.18	8.26	5.57	5.67					
	**H3a**	**H3b**	**H3c**	**H3d**	**H3m1**	**H3m2**					
	5.85	6.53	6.71	7.86	5.38	5.44					

Supplementary Table 5. Observed intermolecular NOE cross-peaks between the 5′ G-tetrad and Pt-tripod. The intensity of the NOE peaks is labeled with ‘S’ (strong), ‘M’ (medium) or ‘W’ (weak).

		Pt-tripod																							
		Segment 1								Segment 2								Segment 3							
		H1a	H1b	H1c	H1d	H1e	H1f	H1m	H1n	H2a	H2b	H2c	H2d	H2e	H2f	H2m	H2n	H3a	H3b	H3c	H3d	H3e	H3f	H3m	H3n
G4	H1	W								M	S	M													
	H8	M	W							S	M							M	M	W					
	H1’									M	W														
	H2’/H2”									W	M	W													
	H3’																								
	H4’									W	W														
	H5’/H5”									M	M							W							
G10	H1	S	S	W						M	M							M							
	H8		W	M	S	M	W	M																	
	H1’			W	M																				
	H2’/H2”																								
	H3’																								
	H4’																								
	H5’/H5”			W	W																				
G18	H1	W	M	M																					
	H8																								
	H1’																								
	H2’/H2”																								
	H3’																								
	H4’																								
	H5’/H5”																								
G22	H1										W	W													
	H8											M													
	H1’												W	M	M	M									
	H2’/H2”																								
	H3’																								
	H4’																								
	H5’/H5”												M												

Supplementary Table 6. Observed intermolecular NOE cross-peaks between the 5′ flanking/loop residues and Pt-tripod. The intensity of the NOE peaks is labeled with 'S' (strong), 'M' (medium) or 'W' (weak).

		Pt-tripod																							
		Segment 1								Segment 2								Segment 3							
		H1a	H1b	H1c	H1d	H1e	H1f	H1m	H1n	H2a	H2b	H2c	H2d	H2e	H2f	H2m	H2n	H3a	H3b	H3c	H3d	H3e	H3f	H3m	H3n
A3	H2	M								S	M							M							
	H8										W	S	M												
	H1'									W	W							S	M						
	H2'/H2''									S	M	M						S	M						
	H3'									W	W														
	H4'																		W						
	H5'/H5''																								
A9	H2	S								M								S	M						
	H8																		W	S	M			W	
	H1'	M	W															M	W						
	H2'/H2''	S	M															S	S	M					
	H3'	W																M	W						
	H4'																								
	H5'/H5''																			W					
A21	H2	S	M	M						S	S	M													
	H8																								
	H1'											M	W												
	H2'/H2''																								
	H3'																								
	H4'												W												
	H5'/H5''																								
T8	H6																				W				
	Me																					M	W		
	H2'/H2''																			W	M				
	H3'																			W	W				
T19	Me						W																		
T20	Me			S	M	W		W																	

Supplementary Table 7. Observed inter-residue NOE cross-peaks at the 5′ binding pocket. The intensity of the NOE peaks is labeled with 'S' (strong), 'M' (medium) or 'W' (weak).

		A21							T20							T19						
		H2	H8	H1'	H2'/ H2''	H3'	H4'	H5'/ H5''	H6	Me	H1'	H2'/ H2''	H3'	H4'	H5'/ H5''	H6	Me	H1'	H2'/ H2''	H3'	H4'	H5'/ H5''
G4	H1	W																				
G10	H1	W																				
G18	H1	S																				
	H8		S	W	S	W																
	H1'															M	M					
	H2'/H2''		M																			
	H3'		M																			
	H4'																M					
G22	H1	M	W	S	M																	
A21	H2								W	M												
	H8								M		M	S	S	W						W		
	H1'											W										
	H2'/H2''																					
	H3'										W											
	H4'										M											
	H5'/H5''										S											
T20	H6	W	M																W	W		
	Me	M																W	M	W		
	H1'		M				M	S														
	H2'/H2''		S	W																		
	H3'		S																			
	H4'		W																			
	H5'/H5''																					
T19	H6																					
	Me																					
	H1'									W												
	H2'/H2''								W	M												
	H3'		W						W	W												
	H4'																					
	H5'/H5''																					

		A9					A3						A2						A1						
		H2	H8	H1'	H2'/ H2''	H3'	H2	H8	H1'	H2'/ H2''	H3'	H4'	H2	H8	H1'	H2'/ H2''	H3'	H5'/ H5''	H2	H8	H1'	H2'/ H2''	H3'	H4'	H5'/ H5''
A9	H2						S		M				W												
	H8																								
	H1'																								
	H2'/H2''																								
	H3'																								
A3	H2	S											M												
	H8													W	M	S	W								
	H1'	M											W												
	H2'/H2''																								
	H3'																								
	H4'															W									
A2	H2	W					M		W											M					
	H8							W											M		M	S	S	M	W
	H1'							M											W						
	H2'/H2''							S				W													
	H3'							W																	
	H5'/H5''																								
A1	H2													M	W										
	H8												M												
	H1'													M											
	H2'/H2''													S											
	H3'													S											
	H4'													M											
	H5'/H5''													W											
T8	H6																								
	Me																								
	H1'		W																						
	H2'/H2''		M																						
	H3'		M																						

Supplementary Table 8. ^{1}H NMR chemical shifts of the 4:2 Pt-tripod–Tel26 complex at 25 ℃.

Tel26	H6/H8	H1/H2	H1'	H2', H2''	H3'	H4'	H5', H5''	NH21, NH22
**G4**		**H3/Me**						
**A1**	7.66	7.73	5.77	2.03, 2.20	4.57	3.97	3.87, 3.46	
**A2**	7.66	7.53	5.64	2.21	4.70	4.10	3.78, 4.06	
**A3**	7.52	7.46	5.62	2.27, 2.14	4.89	4.22	4.07	
**G4**	6.79	11.44	4.89	3.11, 2.56	4.57	3.85	3.64, 3.41	
**G5**	7.58	11.40	5.44	2.32, 2.47	4.80	4.08	3.52, 3.98	6.48, 9.58
**G6**	7.58	10.92	6.24	3.20, 2.79	5.22	4.59	4.22, 3.83	
**T7**	7.77	1.91	6.37	2.30, 2.54	4.71	4.45	4.22, 4.04	
**T8**	7.94	2.04	6.56	2.54	5.03	4.59	4.20, 4.10	
**A9**	8.16	7.92	6.25	2.27	4.78	4.47	4.10	
**G10**	7.39	11.78	5.70	2.67, 3.17	4.72	4.25	3.96, 4.13	6.60, –
**G11**	7.75	12.14	5.42	2.37	4.90	4.25	4.13	5.95, 9.97
**G12**	7.83	11.22	6.11	1.78, 2.33	4.91	4.22	4.14, 3.75	6.71,–
**T13**	7.08	12.60, 1.90	5.28	2.25, 2.31	4.33	4.00	3.08, 3.77	
**T14**	7.10	13.35, 0.70	6.26	1.66, 2.46	5.04	4.44	3.66, 3.97	
**A15**	7.55	7.26	6.26	1.42, 2.24	5.11	4.41	3.08, 3.76	
**G16**	6.83	10.95	5.71	2.25, 2.00	4.85	4.40	4.00, 4.07	
**G17**	7.26	10.75	5.64	2.29, 2.22	4.83	4.13	4.07	6.15, 9.15
**G18**	7.66	11.61	5.99	2.65	5.08	4.39	4.07, 4.12	6.88, –
**T19**	7.76	1.86	6.28	2.33, 2.46	4.87	4.35	4.15, 4.21	
**T20**	6.87	1.07	5.79	0.92, 1.54	4.60	4.07	3.88	
**A21**	7.97	7.41	6.03	2.67, 2.87	4.96	4.37	3.98, 4.14	
**G22**	6.79	10.89	5.81	2.76, 3.35	4.87	4.56	4.07, 4.13	
**G23**	7.87	11.46	5.56	2.40, 2.51	4.92	4.29	4.06, 4.18	5.75, 9.44
**G24**	7.29	10.36	5.81	1.41, 1.94	4.56	4.40	3.77, 3.96	
**A25**	7.44	7.24	5.84	1.91, 2.22	4.79	4.35	3.01, 3.91	
**A26**	7.96	7.66	5.91	2.41, 2.20	4.82	4.54	3.75, 3.94	

	H1a	H1b	H1c	H1d	H1m1	H1m2	He1	He2	Hf1	Hf2	Hn
	6.98	7.58	7.65	8.67	5.98	6.19					
**Pt-tripod (at 5′-end)**	**H2a**	**H2b**	**H2c**	**H2d**	**H2m1**	**H2m2**	3.01	3.26	2.81	2.97	6.80
	6.43	7.28	7.24	8.32	5.74	5.88					
	**H3a**	**H3b**	**H3c**	**H3d**	**H3m1**	**H3m2**					
	5.80	6.63	6.78	7.98	5.43	5.56					

	H1a*	H1b*	H1c*	H1d*	H1m1*	H1m2*	He1*	He2*	Hf1*	Hf2*	Hn*
	6.70	7.27	7.10	8.57	6.09	6.24					
**Pt-tripod (at 3′-end)**	**H2a***	**H2b***	**H2c***	**H2d***	**H2m1***	**H2m2***	2.99	3.23	2.79	2.94	6.72
	6.56	7.89	7.66	8.55	5.66	5.80					
	**H3a***	**H3b***	**H3c***	**H3d***	**H3m1***	**H3m2***					
	6.77	7.64	7.45	8.13	5.49	5.65					

Supplementary Table 9. ^{1}H NMR chemical shifts of the 4:2 Pt-tripod–Tel26 complex at 15 ℃.

Tel26<br>G4	H6/H8	H1/H2<br>H3/Me	H1'	H2', H2''	H3'	H4'	H5', H5''	NH21, NH22
**A1**	7.53	7.60	5.65	1.92, 2.11	4.48	3.87	3.68, 3.35	
**A2**	7.56	7.41	5.50	2.10	4.61	4.01	3.87, 3.95	
**A3**	7.40	7.32	5.49	2.12, 2.01	4.78	4.13	3.98	
**G4**	6.68	11.33	4.77	2.96, 2.45	4.48	3.78	3.57, 3.36	
**G5**	7.50	11.31	5.32	2.21, 2.36	4.71	3.98	3.37, 3.87	6.40, 9.48
**G6**	7.49	10.83	6.15	3.10, 2.71	5.12	4.48	3.91, 3.74	
**T7**	7.67	1.81	6.27	2.21, 2.43	4.60	4.36	4.12, 3.95	
**T8**	7.86	1.94	6.47	2.44	4.93	4.50	4.12	
**A9**	8.07	7.86	6.17	2.14	4.69	4.37	4.00	
**G10**	7.30	11.70	5.60	2.56, 3.06	4.62	4.14	3.85, 4.02	
**G11**	7.65	12.06	5.30	2.25	4.82	4.15	4.02	5.98, 9.93
**G12**	7.77	11.12	6.03	2.45, 1.69	4.82	4.38	4.04, 3.64	6.58, –
**T13**	6.98	12.54, 1.80	5.17	2.13, 2.21	4.22	3.90	2.97, 3.67	
**T14**	7.00	13.29, 0.57	6.15	1.56, 2.37	4.95	4.36	3.55, 3.85	
**A15**	7.45	7.15	6.14	1.32, 2.12	5.00	4.31	2.96, 3.65	
**G16**	6.75	10.86	5.63	1.91, 1.55	4.77	4.32	3.87, 3.96	
**G17**	7.16	10.65	5.55	2.19, 2.12	4.74	4.03	3.97	6.08, 9.14
**G18**	7.58	11.53	5.89	2.54	4.98	4.29	3.98	
**T19**	7.66	1.77	6.18	2.23, 2.36	4.78	4.26	4.06, 4.12	
**T20**	6.79	1.00	5.72	0.86, 1.46	4.51	3.97	3.82	
**A21**	7.89	7.32	5.94	2.77, 2.58	4.86	4.29	3.88, 3.98	
**G22**	6.70	10.82	5.71	2.65, 3.25	4.79	4.47	3.97, 4.04	6.60, 8.55
**G23**	7.78	11.37	5.48	2.35, 2.41	4.83	4.15	3.97, 4.13	5.63, 9.39
**G24**	7.18	10.23	5.70	1.32, 1.90	4.48	4.30	3.68, 3.88	
**A25**	7.34	7.12	5.73	1.81, 2.13	4.70	4.24	2.91, 3.86	
**A26**	7.87	7.53	5.80	2.31, 2.10	4.78	4.44	3.99, 3.90	

**Pt-tripod (at 5′-end)**	**H1a**	**H1b**	**H1c**	**H1d**	**H1m1**	**H1m2**	**He1**	**He2**	**Hf1**	**Hf2**	**Hn**
	6.89	7.49	7.55	8.57	5.92	6.13	2.90	3.15	2.70	2.87	6.74
	**H2a**	**H2b**	**H2c**	**H2d**	**H2m1**	**H2m2**					
	6.34	7.14	7.14	8.21	5.58	5.80					
	**H3a**	**H3b**	**H3c**	**H3d**	**H3m1**	**H3m2**					
	5.69	6.48	6.69	7.87	5.37	5.49					

**Pt-tripod (at 3′-end)**	**H1a***	**H1b***	**H1c***	**H1d***	**H1m1***	**H1m2***	**He1***	**He2***	**Hf1***	**Hf2***	**Hn***
	6.59	7.15	7.00	8.47	6.02	6.14	2.88	3.12	2.68	2.83	6.66
	**H2a***	**H2b***	**H2c***	**H2d***	**H2m1***	**H2m2***					
	6.46	7.79	7.55	8.44	5.59	5.72					
	**H3a***	**H3b***	**H3c***	**H3d***	**H3m1***	**H3m2***					
	6.67	7.53	7.34	8.02	5.40	5.58					

Supplementary Table 10. Observed intermolecular NOE cross-peaks between the 3′ G-tetrad and Pt-tripod. The intensity of the NOE peaks is labeled with ‘S’ (strong), ‘M’ (medium) or ‘W’ (weak).

		Pt-tripod																							
		Segment 1								Segment 2								Segment 3							
		H1a	H1b	H1c	H1d	H1e	H1f	H1m	H1n	H2a	H2b	H2c	H2d	H2e	H2f	H2m	H2n	H3a	H3b	H3c	H3d	H3e	H3f	H3m	H3n
G6	H1									M	M	M													
	H8									W								S	M						
	H1’											M													
	H2’/H2”									W	M	S						S	S						
	H3’																	W	W	W					
	H4’																								
	H5’/H5”																								
G12	H1																								
	H8											W	M			W									
	H1’																								
	H2’/H2”												W												
	H3’												M												
	H4’																								
	H5’/H5”																								
G16	H1		W	S	W																				
	H8																								
	H1’																								
	H2’/H2”																								
	H3’																								
	H4’																								
	H5’/H5”																								
G24	H1	S	M							S	W							M							
	H8			M	S			M																	
	H1’	W	M	W																					
	H2’/H2”	M	S	S	M																				
	H3’			M	S																				
	H4’																								
	H5’/H5”																								

Supplementary Table 11. Observed intermolecular NOE cross-peaks between the 3′ flanking/loop residues and Pt-tripod. The intensity of the NOE peaks is labeled with ‘S’ (strong), ‘M’ (medium) or ‘W’ (weak).

		Pt-tripod																							
		Segment 1								Segment 2								Segment 3							
		H1a	H1b	H1c	H1d	H1e	H1f	H1m	H1n	H2a	H2b	H2c	H2d	H2e	H2f	H2m	H2n	H3a	H3b	H3c	H3d	H3e	H3f	H3m	H3n
T13	H3	M								W	S														
	H6											S	S												
	Me										M	M	S	W	W	W									
	H1’											S	W												
	H2’/H2”																								
	H3’																								
	H4’											M	M												
	H5’/H5”												W												
T14	H3	W	S	M																					
	H6				S	W		W																	
	Me		W	M	S	M	M	W																	
	H1’		W	M	S																				
	H2’/H2”																								
	H3’																								
	H4’				W																				
	H5’/H5”																								
A15	H2																								
	H8	W	M	M	M																				
	H1’																								
	H2’/H2”			M	S																				
	H3’				M																				
	H4’																								
	H5’/H5”																								
A25	H2	W								S	M							S	M						
	H8		M	M														M	W						
	H1’																	M	M	W					
	H2’/H2”																								
	H3’																								
	H4’																		M	W					
	H5’/H5”		W																						

Supplementary Table 12. Observed inter-residue NOE cross-peaks at the 3′ binding pocket. The intensity of the NOE peaks is labeled with ‘S’ (strong), ‘M’ (medium) or ‘W’ (weak).

		T13		T14							A15							A25	
		H3	H1’	H3	H6	Me	H1’	H3’	H4’	H5’/H5”	H2	H8	H1’	H2’/H2”	H3’	H4’	H5’/H5”	H2	H8
G12	H1											W							
	H2’/H2”										M								
G16	H1											W							
	H8												M	S		W			
	H1’													M					
T13	H3																	S	
	H1’										W								
T14	H3																		S
	H6														W		M		
	Me																W		
	H1’											W		W	M	W	S		
	H4’											S	W						
	H5’/H5”											M							
A15	H2																		
	H8						W		S	M									
	H1’								W										
	H2’/H2”						W												
	H3’				W		M												
	H4’						W												
	H5’/H5”				M	W	S												
A25	H2	S																	
	H8			S															

Supplementary Table 13. Observed inter-residue NOE cross-peaks for the A26:A26* pair at the 3′–3′ interface of the 4:2 Pt-tripod-Tel26 dimeric structure. The intensity of the NOE peaks is labeled with ‘S’ (strong), ‘M’ (medium) or ‘W’ (weak).

		T13								T14								A25						
		H3	H6	Me	H1’	H2’/H2”	H3’	H4’	H5’/H5”	H3	H6	Me	H1’	H2’/H2”	H3’	H4’	H5’/H5”	H2	H8	H1’	H2’/H2”	H3’	H4’	H5’/H5”
A26	H2		W	W	W	M	W											M						
	H8									W	W		W	M	M	W	M		S	M	S	W		
	H1’				W	M																		
	H2’/H2”																							
	H3’																							
	H4’																							
	H5’/H5”																							

Supplementary Methods

Synthesis of Pt-tripod:[1] [Pt(dien)Cl]Cl (dien: diethylenetriamine) was synthesized according to literature methods.[2] [Pt(dien)Cl]Cl (110.7 mg, 0.30 mmol) and $AgNO_3$ (101.9 mg, 0.60 mmol) in water (15 mL) were stirred under an inert atmosphere of N_2 in darkness for 40 h at 60 ℃ and then centrifuged to remove AgCl. The clear liquid was subsequently transferred to another round bottom flask and then tri(4-pyridylphenyl)amine) (42.9 mg, 0.09 mmol) was added. The mixture was heated at 90 ℃ for 48 h under N_2 in darkness. The final product was precipitated through the addition of ethanol and then collected by centrifugation, washed with ethanol to obtain a yellow powder. Yield: 121.9 mg (73.2%). ^{1}H-NMR (600 MHz, D_2O): δ = 8.60 (d, J = 6.0 Hz, 6H), 7.76 (m, 12H), 7.30 (d, J = 8.5 Hz, 6H), 3.21-3.27 (dd, J = 14.1, 5.0 Hz, 6H), 2.90-3.05 (m, 12H), 2.79-2.85 (dd, J = 14.1, 5.0 Hz, 6H).

Supplementary References

1. Zhong, Y. F., Zhang, H., Liu, W. T., Zheng, X. H., Zhou, Y. W., Cao, Q., Shen, Y., Zhao, Y., Qin, P. Z., Ji, L. N. & Mao, Z. W. A Platinum(II)-based Photosensitive Tripod as an Effective Photodynamic Anticancer Agent through DNA Damage. *Chem. Eur. J.* **23**, 16442-16446 (2017).
2. F., W. C. & Rudi, v. E. Influence of Solvent on Ligand-Substitution Reactions of PtII Complexes as Function of the -Acceptor Properties of the Spectator Chelate. *Eur. J. Inorg. Chem.* **2005**, 4755-4761 (2005).
3. Dai, J. X., Carver, M., Punchihewa, C., Jones, R. A. & Yang, D. Z. Structure of the Hybrid-2 type intramolecular human telomeric G-quadruplex in K+ solution: insights into structure polymorphism of the human telomeric sequence. *Nucleic Acids Res.* **35**, 4927-4940 (2007).
4. Ambrus, A., Chen, D., Dai, J., Jones, R. A. & Yang, D. Solution structure of the biologically relevant G-quadruplex element in the human c-MYC promoter. Implications for G-quadruplex stabilization. *Biochemistry* **44**, 2048-2058 (2005).
5. Mathad, R. I., Hatzakis, E., Dai, J. X. & Yang, D. Z. c-MYC promoter G-quadruplex formed at the 5 '-end of NHE III1 element: insights into biological relevance and parallel-stranded G-quadruplex stability. *Nucleic Acids Res.* **39**, 9023-9033 (2011).
6. Agrawal, P., Hatzakis, E., Guo, K., Carver, M. & Yang, D. Solution structure of the major G-quadruplex formed in the human VEGF promoter in K+: insights into loop interactions of the parallel G-quadruplexes. *Nucleic Acids Res.* **41**, 10584-10592 (2013).
7. Agrawal, P., Lin, C., Mathad, R. I., Carver, M. & Yang, D. The major G-quadruplex formed in the human BCL-2 proximal promoter adopts a parallel structure with a 13-nt loop in K+ solution. *J. Am. Chem. Soc.* **136**, 1750-1753 (2014).
8. Dai, J., Chen, D., Jones, R. A., Hurley, L. H. & Yang, D. NMR solution structure of the major G-quadruplex structure formed in the human BCL2 promoter region. *Nucleic Acids Res.* **34**, 5133-5144 (2006).
9. Chen, Y., Agrawal, P., Brown, R. V., Hatzakis, E., Hurley, L. & Yang, D. The major G-quadruplex formed in the human platelet-derived growth factor receptor beta promoter adopts a novel broken-strand structure in K+ solution. *J. Am. Chem. Soc.* **134**, 13220-13223 (2012).
10. Dai, J. X., Punchihewa, C., Ambrus, A., Chen, D., Jones, R. A. & Yang, D. Z. Structure of the intramolecular human telomeric G-quadruplex in potassium solution: a novel adenine triple formation. *Nucleic Acids Res.* **35**, 2440-2450 (2007).
