# Supplementary material for: Solution structures of multiple G-quadruplex complexes induced by a platinum(II)-based tripod reveal dynamic binding
Source: Nat Commun. 2018 Aug 29;9:3496. doi: 10.1038/s41467-018-05810-4 (PMC6115404; doi:10.1038/s41467-018-05810-4)
Supplement: Supplementary file 3 — Description of Additional Supplementary Files [file 41467_2018_5810_MOESM3_ESM.pdf]

Description of Additional Supplementary Files

File Name: Supplementary Movie 1
Description: The dynamic binding of hybrid-1 human telomeric G-quadruplex Tel26 and Pt-tripod.
